# Investigating Holocene human population history in North Asia using ancient mitogenomes

**DOI:** 10.1038/s41598-018-27325-0

**Published:** 2018-06-12

**Authors:** Gülşah Merve Kılınç, Natalija Kashuba, Reyhan Yaka, Arev Pelin Sümer, Eren Yüncü, Dmitrij Shergin, Grigorij Leonidovich Ivanov, Dmitrii Kichigin, Kjunnej Pestereva, Denis Volkov, Pavel Mandryka, Artur Kharinskii, Alexey Tishkin, Evgenij Ineshin, Evgeniy Kovychev, Aleksandr Stepanov, Aanatolij Alekseev, Svetlana Aleksandrovna Fedoseeva, Mehmet Somel, Mattias Jakobsson, Maja Krzewińska, Jan Storå, Anders Götherström

**Affiliations:** 1 Department of Archaeology and Classical Studies, Stockholm University, 10691 Stockholm, Sweden; 2 University of Oslo, Museum of Cultural History, 0164 Oslo, Norway; 3 Middle East Technical University, Department of Biological Sciences, 06800 Ankara, Turkey; 4 Laboratory of Archaeology and Ethnography, Faculty of History and Methods, Department of Humanitarian and Aesthetic Education, Pedagogical Institute, Irkutsk State University, Irkutsk, 664011 Irkutsk, Oblast Russia; 5 Irkutsk Museum of Regional Studies, Irkutsk, 664003 Irkutsk Oblast, Russia; 6 Irkutsk National Research Technical University, Laboratory of Archaeology, Paleoecology and the Subsistence Strategies of the Peoples of Northern Asia, Irkutsk State Technical University, Irkutsk, 664074 Irkutsk Oblast, Russia; 7 M. K. Ammosov North-Eastern Federal University (NEFU), Federal State Autonomous Educational Institution of Higher Education, Yakutsk, 677000 Sakha Republic Russia; 8 The Center for Preservation of Historical and Cultural Heritage of the Amur Region, Blagoveshchensk, 675000 Amur Oblast Russia; 9 Siberian Federal University, Krasnoyarsk, 660041 Krasnoyarskiy Kray Russia; 10 The Laboratory of Interdisciplinary Studies in Archaeology of Western Siberia and Altai, Department of Archaeology, Ethnography and Museology, Altai State University, Barnaul, Altaiskiy Kray Russia; 11 Faculty of History, Transbaikal State University, Chita, 672039 Zabaykalsky Kray Russia; 12 The Institute for Humanities Research and Indigenous Studies (IHRISN), Academy of Sciences of the Sakha Republic, Yakutsk, 677000 Sakha Republic Russia; 13 Institute of Arctic Archaeology and Paleoecology, Russian Academy of Sciences, Yakutsk, 677000 Sakha Republic Russia; 14 Department of Organismal Biology and SciLife Lab, Evolutionary Biology Centre, 75236 Uppsala, Sweden

## Abstract

Archaeogenomic studies have largely elucidated human population history in West Eurasia during the Stone Age. However, despite being a broad geographical region of significant cultural and linguistic diversity, little is known about the population history in North Asia. We present complete mitochondrial genome sequences together with stable isotope data for 41 serially sampled ancient individuals from North Asia, dated between c.13,790 BP and c.1,380 BP extending from the Palaeolithic to the Iron Age. Analyses of mitochondrial DNA sequences and haplogroup data of these individuals revealed the highest genetic affinity to present-day North Asian populations of the same geographical region suggesting a possible long-term maternal genetic continuity in the region. We observed a decrease in genetic diversity over time and a reduction of maternal effective population size (N_e_) approximately seven thousand years before present. Coalescent simulations were consistent with genetic continuity between present day individuals and individuals dating to 7,000 BP, 4,800 BP or 3,000 BP. Meanwhile, genetic differences observed between 7,000 BP and 3,000 BP as well as between 4,800 BP and 3,000 BP were inconsistent with genetic drift alone, suggesting gene flow into the region from distant gene pools or structure within the population. These results indicate that despite some level of continuity between ancient groups and present-day populations, the region exhibits a complex demographic history during the Holocene.

## Introduction

**Figure 1:**
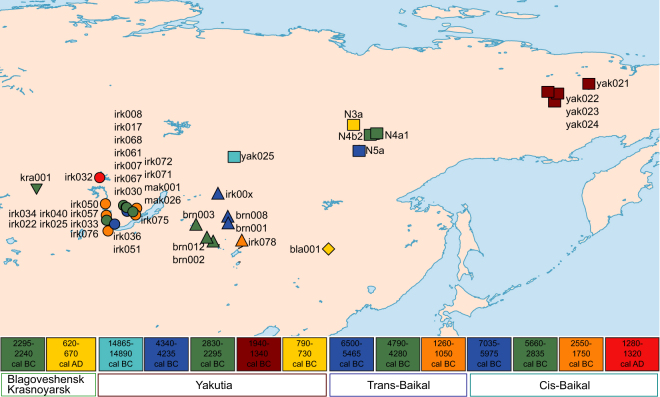
Geographic map and timeline showing the sampling locations and radiocarbon dated ages of ancient individuals. For detailed information about the samples and sites, authenticity of the sequences and stable isotopes see Supplementary Tables S1–S3 and Figs S1 and S2 in Supplementary Information. Cis-Baikal individuals are represented as circles, Trans-Baikal individuals are represented as triangles and Yakutia individuals are represented as squares.

Recent ancient DNA studies have contributed to four major discoveries about the Holocene human population history in Eurasia: i. Gene flow from Near East through Europe during the Neolithization^1–7^, ii. Genetic continuity between pre-Neolithic and Neolithic populations of Near East^8–10^, iii. Increased mobility in West Eurasia during Bronze Age^11,12^, and iv. Genetic continuity in East Asia during the Holocene^13^. In contrast, the population history in North Asia has remained largely unknown with a limited number of published ancient genomes^14,15^. Here we fill this archaeogenetic gap by examining complete mitochondrial genome sequences and presenting radiocarbon dates of 41 serially sampled ancient individuals from North Asia, corresponding to the three major administrative regions of the Russian Federation including Cis-Baikal (Irkutsk Oblast), Trans-Baikal (Republic of Buryatia and Zabaykalsky Krai) and Yakutia (Sakha Republic) (Fig. 1 and Supplementary Table S1).

Archaeological data indicates an intensive and complex prehistory in North Asia. East Siberia has been inhabited since the early Paleolithic, as evidenced by sites with pebble industries^16–19^. The first humans populating the region had West Eurasian origin^20,21^. The Baikal region of Siberia has been occupied by humans since Middle Palaeolithic^22,23^. The areas west (Cis-Baikal) and east (Trans-Baikal) of the Lake Baikal have been inhabited since the Palaeolithic and together with Yakutia exhibit a vast variety of prehistoric cultures, including the Neolithic and Bronze Age Kitoi and Glazkovo cultural entities^16,18,24–26^. Both archaeological and genetic data have shown similarities between the cultures of Cis-/Trans-Baikal regions and Yakutia^27–31^. The Neolithic in North Asia is not associated with sedentism and agriculture, but characterized by the appearance of characteristic stone production techniques (i.e. polishing) and presence of pottery of eastern origin^26^. Until the Iron Age, the region was inhabited by foraging groups. The most important cultural shift in the region might be associated with the arrival of metal in the Bronze Age as well as beginning of pastoralism in the Iron Age^32^.

## Results

### Ancient mitochondrial genome sequencing and stable isotope analysis

We generated complete mitochondrial genome sequences of 41 ancient individuals with coverages between 12× and 357× (median = 60×) excavated from the Baikal and Yakutia regions in North Asia (Fig. 1, and Supplementary Table S2). 14 individuals were genetically identified as females and 27 were males (Supplementary Table S2)^7^. All libraries showed elevated frequencies of cytosine deamination at 5′ read termini^33^ (Fig. S1 in Supplementary Information). Point estimates of contamination ranged between 0–25% (95% CI of 0–35%) based on mitochondrial genetic variation^34^ (Supplementary Table S3). Bayesian mitochondrial contamination estimates^35^ ranged between 0–11% (Supplementary Table S3). Evaluating possible maternal kinship among individuals buried in the same location, we identified three potential maternal kinship cases in our dataset. These comprised a Late Neolithic triple burial (Haplogroup C4b, individuals yak022, yak023 and yak024) from Kamenka 2 (Kolyma river), a Bronze Age double burial (Haplogroup D4j, individuals irk071, irk072) from Mys Uyuga (Irkutsk Oblast) and two Late Neolithic individual burials from Kyordyughen, (Central Yakutia) (Haplogroup A12a, individuals N4a1 and N4b2).

Radiocarbon dating on osteological material from 37 of 41 individuals using accelerator mass spectrometry (AMS) placed the material between c.13,790 BP and c.1,380 BP (Supplementary Table S2 and Fig. S2a,b,c and d in Supplementary Information). To analyse the variation in the diet and subsistence practices of these individuals we examined the stable isotope values (δ^13^C and δ^15^N) (Supplementary Table S2). Almost all individuals had elevated δ^15^N and lower δ^13^C values suggesting an aquatic diet (Fig. S2e,f and g in Supplementary Information), consistent with archaeological evidence supporting an important fresh-water fish consumption in the region^36^.

### Mitochondrial DNA haplogroup- and sequence-based analyses reveal genetic similarities between ancient and modern North Asians

We identified 25 different mtDNA haplogroups across all individuals (Supplementary Tables S4 and S5), belonging to the macro-haplogroups M, N, or R. These three non-African macro-haplogroups have been reported to have diverged around 60–65 kya, and being carried to Southeast Asia by the first modern humans^37^. Specifically, 38 individuals carried the East Eurasian mitochondrial haplogroups of A, C, D, F, G and their sub-haplogroups. A Palaeolithic individual from Yakutia and a Bronze Age individual from Cis-Baikal carried the mitochondrial haplogroups R1 and R1b, respectively (Supplementary Table S4); which are sub-clades of the common West Eurasian macro-haplogroup R that was also observed in the Upper Paleolithic Ust’-Ishim^14^. As the Ust’ Ishim is from West Siberia, our result raises the possibility that the R haplogroup may have been distributed throughout North Asia.

**Figure 2:**
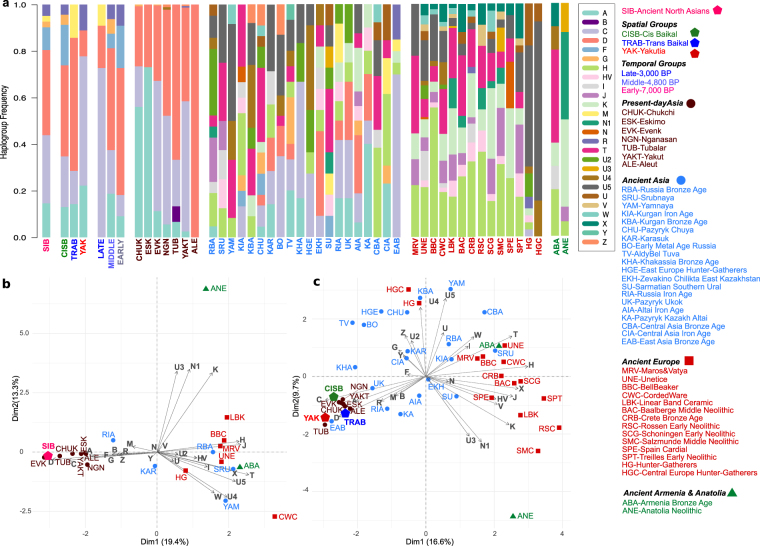
Relationship between ancient North Asians and other populations based on haplogroup frequencies. Ancient North Asians as a single group (SIB, n = 41) and as divided into three different regional groups including Cis-Baikal (CISB, n = 23), Trans-Baikal (TRAB, n = 7) and Yakutia (YAK, n = 9) or as divided into three temporal groups including Early (7,000 BP, n = 11), Middle (4800 BP, n = 16) and Late (3000 BP, n = 11). Two individuals from Krasnoyarsk and Blagoveshensk are not included in regional groups due to their distinct geographical locations. (**a**) Barplot showing haplogroup frequencies on a dataset of 1,780 individuals. PCA plot based on haplogroup frequencies calculated using (**b**) 291 individuals with full mitochondrial sequences. Ancient North Asians are included as a single population. (**c**) 1,780 individuals. Ancient North Asians are included as three different regional groups in the analysis. See also Supplementary Tables S1, S4–S12 and Fig. S3a and b in Supplementary Information.

To assess the maternal genetic relationship with other ancient and present-day populations, we compiled two haplogroup frequency datasets by merging 41 ancient individuals with ancient and present-day individuals i. comprising full mitochondrial sequences (n = 291), and ii. comprising full mitochondrial sequences, mitochondrial HVRI (16059–16365) sequences and haplogroup data (n = 1,780) (Supplementary Tables S6–S8). Haplogroup frequencies were calculated by grouping ancient North Asians into (a) a single group, (b) three spatial groups (Cis-Baikal (CISB, n = 23), Trans-Baikal (TRAB, n = 7), and Yakutia (YAK, n = 9)) and (c) three temporal groups (Early (n = 11, mean = 7,000 BP), Middle (n = 16, mean = 4,800 BP), and Late (n = 11, mean = 3,000 BP)) (Supplementary Tables S9–S11). This analysis revealed that the haplogroup distribution in ancient North Asians is similar to that of present-day populations of the same region (Fig. 2a). Principal component analysis (PCA) of the haplogroup frequency data based on full mitochondrial genome sequence dataset revealed that ancient individuals grouped as a single unit clustered with present-day populations of the same region, to the exclusion of other ancient groups (Fig. 2b). This was also observed when more population groups were included in the analysis (Fig. S3a in Supplementary Information) and when ancient individuals were spatially (Fig. 2c) or temporally (Fig. S3b in Supplementary Information) grouped. This lack of distinction between ancient and present-day groups could, however, be resulted from relative homogeneity of the haplogroup variation amongst North Asian groups^38^.

**Figure 3:**
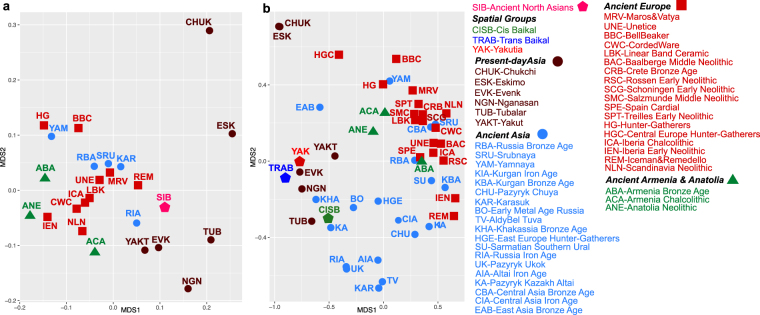
Relationship between ancient North Asians and other ancient and present-day populations based on Slatkin’s linearized pairwise *F*_*ST*._ MDS plot based on Slatkin’s linearized pairwise *F*_*ST*_ calculated using (**a**) full mitochondrial DNA sequences. (**b**) HVRI sequences. See also Fig. S3c and d in Supplementary Information, Supplementary Tables S13–S15.

Since the haplogroup frequency based analysis might be affected by relatively low sample size of some populations, we further evaluated the genetic affinities between ancient North Asians and other populations based on the mitochondrial sequences. We calculated Slatkin’s linearized pairwise *F*_*ST*_ on two datasets including ancient and present-day individuals i. with full mitochondrial sequences (n = 355) and ii. with HVRI sequences (n = 1,140) both merged with presented ancient individuals from North Asia (Supplementary Tables S12 and S13). We observed low *F*_*ST*_ between ancient and modern North Asian populations including Evenk, Nganasan and Tubalar (Fst ≤ 0.05) (Fig. S3c and d in Supplementary Information, Supplementary Table S14). MDS analysis based on *F*_*ST*_ showed that the first dimension differentiated both the present-day and the ancient North Asians from other ancient groups (Fig. 3a). We observed consistent results even when more population groups were included in the analysis and when the ancient North Asian individuals were grouped into three different spatial populations (Fig. 3b and Supplementary Table S14).

Although highly dependent on sample size and thus prone to generalization, haplotype sharing analysis between three spatial groups and other modern and ancient populations (Supplementary Table S15) revealed that the TRAB group shared most lineages with ancient Kazakh Altai (KA) and modern Nganasan (NGN)^39–42^. The CISB group shared most lineages with Tubalar^39,42^, KA^43^ and Early Bronze Age groups of Russia (BO)^12^, which might reflect the Siberian roots of BO, consistent with MDS based on *F*_*st*_ (Fig. 3b). The YAK group shared most lineages with the CISB, BO and Tubalar groups. These results showed that despite being from different sides of the Lake Baikal, the CISB and YAK groups shared most lineages with the Tubalar and also both of them were to a certain degree affiliated to the BO of the Cis-Baikal region, thus, reflecting a shared common ancestry. Furthermore, the CISB and YAK groups share lineages supporting the hypothesis of a lasting continuity in this large geographical territory. However, the TRAB group may have different legacy with affinities to ancient Kazakh Altai and modern Nganasan groups (that, actually, may have relocated from the Trans-Baikal region in times post-dating our sample).

### Assessing maternal genetic diversity and population size change and testing population continuity

We calculated haplotype diversity using the dataset comprising full mitochondrial sequences and by grouping all individuals into a single population which reveals high maternal genetic diversity in the whole region (0.994 ± 0.007). Since the individuals were sampled from a large region and a long time period, we further evaluated the haplotype diversity in the spatial (CISB, TRAB, YAK) and in the temporal (Early, Middle, Late) groups. The range of haplotype diversity in the spatial groups (0.917–1, median = 0.992) was similar to that in the temporal groups (0.945–1, median = 0.992) (Mann-Whitney U-test p > 0.05). Although temporal grouping revealed that genetic variation decreased over time in the Lake Baikal and Yakutia regions (0.945–1, median = 0.992), diversity estimates for both spatial and temporal groups were not significantly lower than for the present-day populations in the region (0.964–1, median = 0.969) (Mann-Whitney U-test p > 0.05, assuming independence among populations) (Supplementary Table S16).

**Figure 4:**
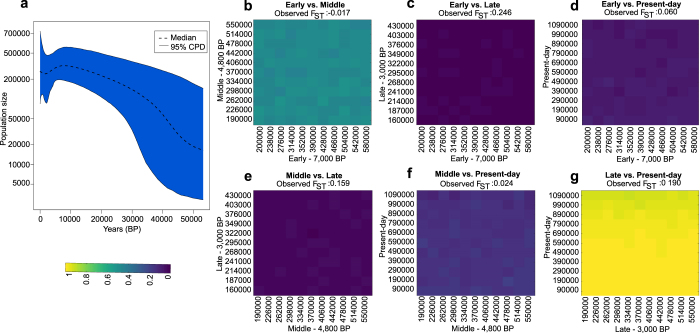
Assessment of past maternal effective population size and testing regional population continuity by coalescent simulation. (**a**) Extended Bayesian Skyline plot of maternal effective population size history on all ancient North Asians with radiocarbon ages together with modern populations from the same geographical region based on generation time of 25. Strict clock model was used. For clock rate, we assumed that the generation time for humans is 25 years, and the mutation rate is 3.4 × 10^−7^ per site per generation for the full human mitochondrial genome sequence^77^. Radiocarbon ages for each of ancient individuals were used as tip dates for molecular clock calibration. MCMC chain was run for 100 million steps. (**b**–**g**) The grids represent results of 11 × 11 × 1000 serial coalescent simulations under the exponential growth model for six comparisons between North Asian ancient individuals grouped into Early (7,000 BP), Middle (4,800 BP), Late (3,000 BP) and present-day groups. The effective population sizes used in the simulations are shown on the x- and y-axes. The colors indicate the proportion of 1000 simulations in each grid that had *F*_*ST*_ values greater than that observed *F*_*ST*_. Comparisons include (**b**) Early vs. Middle, (**c**) Early vs. Late, (**d**) Early vs. Present-day, (**e**) Middle vs. Late, (**f**) Middle vs. Present-day, and (**g**) Late vs. Present-day. See also Supplementary Tables S17 and S18.

We examined the maternal effective population size history in the region by employing Extended Bayesian Skyline Plot (EBSP) analysis using BEAST^44^ (Supplementary Table S17). EBSP analysis revealed increasing maternal effective population size between 50,000–7,000 BP followed by a decrease approximately around 7,000 BP (Fig. 4a). We formally tested population continuity using a total of 117 individuals (38 ancient and 79 present-day) with full mitochondrial genome sequences (Supplementary Table S18), by grouping the ancient individuals into three temporal groups based on their radiocarbon dates. Here we test the null hypothesis of continuity, specifically, that two diachronic populations we sampled belonged to a single resident population that diverged by genetic drift only. To test this, we conducted population genetic simulations under different demographic scenarios, and asked whether the observed differentiation between three temporally-divided groups and present-day populations in the region can be explained by genetic drift within a given time interval, assuming an exponential growth model, and a wide range of population sizes. If most of the simulations yield smaller *F*_*ST*_ than observed, we reject the null hypothesis, which is an indication of gene flow that caused differentiation in time between the two populations, or that the two populations sampled did not belong to the same regional population, i.e. population structure (Fig. 4b–g and Supplementary Table S18). When we compared Early vs. Middle, Early vs. present-day, Middle vs. present-day, and Late vs. present-day populations, genetic differentiation was found to be modest (*F*_*ST*_ < 0.10, except for the Late vs. present-day comparison). The 121,000 simulations conducted for each comparison frequently (>95%) yielded *F*_*ST*_ values equal to or higher than those observed (Fig. 4b,d,f and g). We therefore cannot reject the null hypothesis that these diachronic samples derived from a single resident maternal gene pool in the region. However, in comparisons involving Early vs. Late, and Middle vs. Late populations, *F*_*ST*_ was >0.15, and the simulations conducted rarely (<5%) yielded as large *F*_*ST*_ values across all 11 × 11 demographic parameter combinations studied in each comparison (Although was not significant after multiple testing correction) (Fig. 4c and e). Hence, differences between the Late population sample and both earlier and later-coming groups cannot be explained by genetic drift alone, and the Late population sample may not belong to the same residential population as the others.

## Discussion and Conclusion

Lake Baikal and Yakutia have a rich Holocene archaeological record allowing the investigation of population history, demographic events, and adaptation to the environment in North Asia^32^. In this work, we present the stable isotopes of carbon and nitrogen as well as mitochondrial genomes of 41 ancient individuals from the Lake Baikal and Yakutia belonging to the general chronological frame of the North Asian archaeological cultural complexes^32,45–47^.

Dietary reconstruction through carbon and nitrogen stable isotope analysis provides important insights into subsistence strategies of human populations as well as their adaptation to the environment. In general, carbon and nitrogen stable isotopes of the skeletal samples from western, eastern, and northern shores of the Lake Baikal and from Yakutia revealed that protein source in the diet of those individuals was mainly based on aquatic resources, consistent with previous studies^48^. The variation in both the δ^13^C and δ^15^N values in the present study showed that there were differences in the amount of consumed aquatic resources, and probably also differences in the consumed fish species between the individuals and groups, especially between different geographical regions. For example, Yakutia individuals had the lightest δ^13^C values (Supplementary Table S2), which might indicate the consumption of open- and deep- water fish species. However, we observed that the range of δ^13^C values of the individuals from the region around the Lake Baikal included the shallow water as well as open-water fish species, which has been observed earlier^36,48^.

Examination of the mitochondrial genomes led to the discovery of the West Eurasian R1 haplogroup in two individuals. This finding raised the possibility of a common distribution of this haplogroup during the Holocene in a large region of Asia. This was supported also by the presence of the mitochondrial haplogroup F1b (descending from R9) in Mesolithic and Early Neolithic Cis-Baikal individuals (Supplementary Table S4). The mitochondrial haplogroups F and R are widespread amongst modern southern and eastern Asian groups^39,49–51^ and the presence of R in Asia has been considered a remnant of the earliest human expansions in the continent^52^. Presence of the R1 haplogroup in the Palaeolithic individual from Yakutia supported the archaeological records pointing to the West Eurasian origin^20,21^ of the first humans in the region. Furthermore, presence of East Eurasian mitochondrial haplogroups amongst the Neolithic individuals supported the eastern origin of pottery in the region^26^.

The observation of the haplotype sharing between the CISB and TRAB groups, and between the CISB and YAK groups might imply a possible regional maternal genetic continuity during the Holocene. Additionally, our population genetic simulations generally supported continuity, i.e. differentiation only due to genetic drift, within the last ten thousand years.

Two findings, however, were intriguing. One was the discovery of only weak support for a single regional population in comparisons between Early vs. Late as well as Middle vs. Late groups in the region. This may be explained by population structure, as the Late group comprised geographically very distant individuals, such as individuals from Krasnoyarsk Krai and Amur Oblast, not represented in the other diachronic groups (Table S9). Another explanation for rejecting the null hypothesis of continuity between the Middle and Late (4,800–3,000 BP) groups might be due to an interruption and the arrival of pastoralists at the beginning of the Iron Age between 3,670 to 2,760  BP as suggested by the archaeological record^32^. Thus, the introduction of the new lifeways, technologies and material culture expressions might also here be associated to an increased mobility into the area.

The second point was the estimated reduction in maternal effective population size and haplotype diversity around 7,000 BP. Intriguingly, climate modelling and radiocarbon dating studies^53^ suggest that climatic change and a collapse of the riverine ecosystems might have affected the human populations in Cis Baikal between 7,000–6,000 BP in line with our results. This finding was further supported by archaeological studies pointing to a possible hiatus^38,54,55^.

Although our results provide a first glimpse into population structure and diversity in North Asia during the Holocene which link to trend in the archaeological record, complete genome sequences will provide a higher resolution of more complex demographic events in the region.

## Methods

### Ancient DNA extraction, library building and sequencing

Sample preparation, extraction of DNA from bone and teeth samples and library preparation were carried out in ancient DNA facilities at the Archaeological Research Laboratory (AFL), Stockholm University. The samples were cleaned from earth and potential contaminants and the teeth were wiped with 1–3% NaOCl solution and ddH_2_O, and were thereafter subjected to UV radiation at about 6 J/cm^2^ at 254 nm. Bone powder or bone fragments were obtained using a Dremel drill. Weight of powder or fragment ranged from 50 mg to 400 mg. The samples were digested in 1 M Urea, EDTA (0.5 M) and Proteinase K (10 mg/ml)^56^, and after full disolution the DNA extract was concentrated using Amicon filters (Millipore) and purified with silica-based MinElute spin columns (Qiagen)^5,57^. A blank negative control was added for every 8–10 samples in the isolation step. The DNA was eluted in 110 ul of Elution Buffer (EB) (Qiagen). Double-stranded blunt-end Illumina genomic libraries were prepared from 20 µl DNA following Meyer & Kircher protocol^58^ with omission of sonication step unnecessary when handling aDNA. Each library was amplified in five replicates, using 0.5 ul of 10 µM index primer per library, following the thermocycling conditions as in^59^ with the number of cycles estimated individually for each library based on quantitative PCR (qPCR). For sample N4b2DR, three damage-repaired libraries were prepared with USER enzyme (NEB/BioNordika). Libraries were sequenced using Illumina HiSeq X platform at the SciLife sequencing center at Stockholm.

### Sequence data processing

Sequence data was analysed as in^59,60^. In brief, paired-end reads with a minimum 11 bp overlap were merged and residual adapter sequences were trimmed^61^. Merged reads were mapped to the human reference genome (version hs37d5) using BWA^62^ version 0.7.13 in single end mode with parameters “−n 0.01 −o 2 −l 16500”^6^. PCR duplicates were filtered using FilterUniqSAMCons_cc.py^61^. Reads mapped to the reference genome with more than 10% mismatches and shorter than 35 bp were filtered. To prepare comparative dataset, we remapped published ancient genome sequence data for individuals labeled as “samples published fullMT seq” in Supplementary Tables S4 and S5 using the same procedure.

### Assessment of the authenticity

Ancient DNA specific nucleotide misincorporation patterns (increased frequency of cytosine to thymine transitions at 5′ end of DNA)^33^ were examined using PMDtools^6^. Mitochondrial DNA based contamination estimation was performed following a method developed in^34^ and a Bayesian method developed in^35^ as in^60^. In the method based on Green *et al*., alleles present less than 5% in 311 modern mtDNAs (private or near private consensus alleles) with depth of minimum 10× and base quality of minimum 30 were identified. After filtering transition type mutations, point estimate of contamination was obtained by summing the counts of consensus and alternative alleles across all sites. In the method based on Fu *et al*., mitochondrial consensus sequences were called per sample using samtools mpileup^63^ and vcftools^64^. Using “contamMix” library in R, probabilities of authenticity were calculated.

### Biological sex determination

Biological sex of the individuals was determined by Ry method^7^. Analysis was restricted with reads with minimum mapping quality of 30. Ratio of reads mapping to Y chromosome to the reads mapping to both X and Y chromosome was calculated.

### Assignment of the haplogroups

To determine the mitochondrial haplogroups of the individuals sequenced in this study (n = 41) and of the published individuals for which full genome sequences were available (ancient, n = 205) (Supplementary Tables S4–S6) consensus mitochondrial sequences were produced in fasta format using bam files as input. To call the consensus, ANGSD^65^ was used with parameters “-doFasta 2 -doCounts 1 -minQ 30 -minMapQ 30 -setMinDepth 3”. For a total of 115 present-day individuals (Supplementary Table S8), full mitochondrial sequence data were retrieved from GenBank. Mitochondrial haplogroups were initially determined using both Haplofind^66^ and HaploGrep2^67^. Final assignment of the haplogroups was done based on visual inspection against PhyloTree mtDNA tree (build 17)^68^. For 667 ancient individuals, mitochondrial HVRI region sequences (16059–16365) were obtained from GenBank. Mitochondrial haplogroups of these individuals were assigned comparing informative SNPs in HVRI region which are reported against revised Cambridge Reference Sequence (CRS)^69,70^ to PhyloTree mtDNA tree (build 17)^68^ (Supplementary Tables S4–S6). For additional 822 present-day individuals, haplogroup data were retrieved from publications (Supplementary Table S8).

### Biostatistical analysis

Mitochondrial haplogroup frequencies and 95% confidence intervals were calculated on two different datasets (Supplementary Tables S10 and S11) using a Bayesian method (http://www.causascientia.org/math_stat/ProportionCI.html). Principal component analysis (PCA) was performed using “PCA” function of “FactoMineR” library in R. “factoextra” library in R was used to extract and visualize the eigenvalues and variances of the dimensions. To measure genetic differentiation among populations, Slatkin’s linearized *F*_*ST*_ between pairs of populations^71^ was calculated and the significance of the *F*_*ST*_ was assessed by 10,000 random permutations of population labels using Arlequin v.3.5.2^72^. *F*_*ST*_ was applied to two datasets including samples with full and partial (HVRI, 16059–16365) mtDNA sequences (Supplementary Tables S12–S14). Prior to *F*_*st*_ calculation, multiple sequence alignment was performed for both datasets using the ClustalW^73^ and the overlapping region of mtDNA HVRI sequences across all the populations were determined to be 307 bp in length. Non-metric multidimensional scaling (NMDS) on *F*_*ST*_ values was performed using the “metaMDS” function in the “vegan” library and plotted using “ggplot2” library in R. Mitochondrial haplotype diversity, the probability that two randomly chosen haplotypes are different in the sample^74^, was calculated for all population samples using DnaSP^75^ v.6 by “excluding sites with missing data” option. Haplotype sharing was calculated using Arlequin v.3.5.2^72^. The normalized haplotype sharing was estimated by dividing the number of shared haplotypes between two groups divided by the maximum number of possible comparisons (Nmax = Ntot*(Ntot − 1)/2).

### Reconstruction of maternal effective population size

To infer the history of female effective population size change, extended Bayesian Skyline Plot method^76^ was performed on all ancient North Asians using BEAST^44^. The method was applied on a dataset consisting of 37 ancient North Asians with known ^14^C ages together with modern individuals from North Asia with full mitochondrial sequences (Supplementary Table S17). Using ClustalW^73^, multiple sequence alignment of complete mitochondrial DNA sequences was performed. The sequences were partitioned as protein coding and non-coding HVRI. Strict clock model with was used. For clock rate, we assumed that the generation time for humans is 25 years, and the mutation rate is 3.4 × 10^−7^ per site per generation for the full human mitochondrial genome sequence^77^. Tree models and clock models were linked for each partition. Radiocarbon ages for each ancient individual was used as tip dates for molecular clock calibration. MCMC chain was run for 100 million steps. Log file produced by BEAST was inspected using Tracer. EBSP plot was generated using PlotEBSP function in R.

### Coalescent simulation analysis

Population continuity was tested by calculating the observed *F*_*ST*_ for specific pairs of ancient and modern-day populations from the region (Supplementary Table S18) and comparing this with the simulated *F*_*ST*_ values generated by coalescent simulations. Simulated DNA samples for the populations (modern-day, ancient and more ancient) were generated by coalescent simulations using the fastsimcoal2 software^78^. Here, we tested the null hypothesis that the observed *F*_*ST*_ value could arise by genetic drift (*i*.*e*. population continuity). We sampled simulated populations through time, with a demographic scenario of exponential population growth, employing a range of parameter values such as different effective population sizes (Supplementary Table S18). Effective population size of each population was determined based on the Extended Bayesian Skyline Plot (EBSP) analysis result. The exponential growth rate was calculated as the natural logarithm of the ratio of two tested population sizes. We assumed that the generation time for humans is 25 years, and the mutation rate is 3.4 × 10^−7^ per site per generation for the full human mitochondrial genome sequence^77^. We performed 1000 simulations for each combination of population sizes, computed *F*_*ST*_ using Arlequin v.3.5.2^72^ and calculated the proportion of simulated *F*_*ST*_ values that are greater than the observed *F*_*ST*_. Details about the simulation parameters, population datasets, *F*_*ST*_ values and each pairs of population comparisons are given in Supplementary Table S18.

### Data availability

The new generated full mitochondrial genome data have been deposited in GenBank under ID code: MH359189 - MH359229.

## Electronic supplementary material

Supplementary Information

Dataset 1

Dataset 2
