# Supplementary material for: Investigating Holocene human population history in North Asia using ancient mitogenomes
Source: Sci Rep. 2018 Jun 12;8:8969. doi: 10.1038/s41598-018-27325-0 (PMC5997703; doi:10.1038/s41598-018-27325-0)
Supplement: Supplementary file 1 — Supplementary Information [file 41598_2018_27325_MOESM1_ESM.pdf]

Investigating Holocene human population history in North Asia using ancient mitogenomes

Gulsah Merve Kilinc[1*], Natalija Kashuba[1,2*], Reyhan Yaka[3*], Arev Pelin Sumer[3], Eren Yuncu[3], Dmitrij Shergin[4], Grigorij Leonidovich Ivanov[5], Dmitrii Kichigin[6], Kjunnej Pestereva[7], Denis Volkov[8], Pavel Mandryka[9], Artur Kharinskii[6], Alexey Tishkin[10], Evgenij Ineshin[4], Evgeniy Kovychev[11], Aleksandr Stepanov[7], Aanatolij Alekseev[12], Svetlana Aleksandrovna Fedoseeva[13,14], Mehmet Somel[3], Mattias Jakobsson[15], Maja Krzewińska[1], Jan Storå[1], Anders Götherström[1]

[1] Department of Archaeology and Classical Studies, Stockholm University, 10691 Stockholm, Sweden. [2]University of Oslo, Museum of Cultural History, 0164, Oslo.[3]Middle East Technical University, Department of Biological Sciences, 06800, Ankara, Turkey. [4]Laboratory of Archaeology and Ethnography, Faculty of History and Methods, Department of Humanitarian and Aesthetic Education, Pedagogical Institute, Irkutsk State University, Irkutsk, 664011, Irkutsk Oblast, Russia. [5]Irkutsk Museum of Regional Studies, , Irkutsk, 664003, Irkutsk Oblast, Russia. [6]Irkutsk National Research Technical University, Laboratory of Archaeology, Paleoecology and the Subsistence Strategies of the Peoples of Northern Asia, Irkutsk State Technical University, Irkutsk, 664074, Irkutsk Oblast, Russia. [7]M. K. Ammosov North-Eastern Federal University (NEFU), Federal State Autonomous Educational Institution of Higher Education, Yakutsk, 677000, Sakha Republic, Russia. [8]The Center for Preservation of Historical and Cultural Heritage of the Amur Region, Blagoveshchensk, 675000, Amur Oblast, Russia. [9]Siberian Federal University, Krasnoyarsk, 660041, Krasnoyarskiy Kray, Russia. [10]The Laboratory of Interdisciplinary Studies in Archaeology of Western Siberia and Altai, Department of Archaeology, Ethnography and Museology, Altai State University, Barnaul, Altaiskiy Kray, Russia. [11]Faculty of History,

Transbaikal State University, Chita, 672039, Zabaykalsky Kray, Russia. [12]The Institute for Humanities Research and Indigenous Studies (IHRISN), Academy of Sciences of the Sakha Republic, Yakutsk, 677000, Sakha Republic, Russia. [13]Deceased. [14]Institute of Arctic Archaeology and Paleoecology, Russian Academy of Sciences, Yakutsk, 677000, Sakha Republic, Russia. [15]Department of Organismal Biology and SciLife Lab, Evolutionary Biology Centre, Norbyvägen 18 A, SE-752 36 Uppsala, Sweden. Correspondence and requests for materials should be addressed to G.M.K. (email: gulsahhdal@gmail.com) or A.G. (email:anders.gotherstrom@arklab.su.se).

Supplementary Information

Our sample set comprise of 41 North Asian individuals from 34 different sites (**Fig. 1**), chronologically stretching over the period from the Upper Paleolithic and up to Medieval period. The chronology is here based on a total of 37 samples submitted for AMS radiocarbon dating (**Supplementary Tables S1 and S1)**. The four samples without radiocarbon date (Irk030, irk040, Irk068, yak024) have reliable archaeological contexts to securely be given broad chronological intervals. The Paleolithic individual (yak025) was recovered in the Khaiyrgas Cave in Yakutia and is a valuable addition to known Paleolithic humans from Eurasia, **Fig. S2d.** The majority of individuals in our study are dated to Neolithic and Bronze Ages. Only a few are of Mesolithic, Iron Age and Medieval. Some of the ancient individuals are from previously unpublished burial sites. All analysed individuals may be grouped according to a general chronological frame of North Asian archaeological cultural complexes [1–9]. In the comparisons the analysed prehistoric individuals are grouped into three chronological groups; an Early (mean age = 7,000 BP), a Middle (mean age = 4,800 BP), and a Late group (mean age = 3,000 BP) **(Supplementary Table S9)** that all were compared to present-day individuals (mean age= 0) as well as three regional groups; Cis-Baikal, Trans-Baikal and Yakutia. Below we present a description of the archaeological contexts of the studied individuals.

Cis-Baikal group

The topographical term Cis-Baikal is used to describe the region west and north of Baikal lake. The subareas are the Upper Angara river Valley, the Upper Lena river Valley, and lake Baikal, i.e. corresponding to the “micro regions” often referred to in archaeological research [1,7,10,11]. The Cis-Baikal area exhibits a large number of well-documented archaeological sites, from 21 of which we received anthropological material [1,8,9,11–16].

The cultural and chronological schemes of the Cis-Baikal territory are based mostly on observations from burial complexes, extensive use of which resulted in layering and overlapping of the burials belonging to different cultures (e.g. in Glazkovo site). The chronologies and cultural affinities were partially resolved by the archaeological studies based on radiocarbon analysis on anthropological material from Siberia [11,17]. In the context of Baikal Archaeological Project, the horizon of radiocarbon dates of Cis-Baikal has been widened. Currently, all burials and cemeteries of Cis-Baikal are receiving radiocarbon dates and the databank contains radiocarbon results for more than 900 individuals [17–19]. These studies provided better understanding of the absolute chronology of the "archaeological cultures", i.e. which of them actually were contemporaneous and co-existed in time and region and which that were more distinct complexes [18]. The economy of the ancient population of North Asia and the Neolithic transition are themes for future research.

The Neolithic in North Asia is defined by the appearance and dispersal of new stone (tool) technologies (nephrite) and the use of different manufacturing methods [19–21]. Important features of the period are several categories of tools, which include the bow, early pottery and fishing equipment of great variety: fishing hooks (often of composite types) and harpoons [10]. The earliest known pottery in Eurasia is found in South-East China 20,000 - 16,000 BP [22,23]. In North Asia the oldest ceramic dates are from 14,000 – 10,000 BP and are found in the Transbaikal region, the lower Amur river, in Primorsky Krai and in Japan [20,24]. Early pottery has also been discovered in the Cis-Baikal area but the remains a rare type of archaeological artefact [10]. Pottery has been found on a number of sites examined in the present study. For instance, remains of pottery were found in Popovskij Lug Popovskij Lug in layers dated to the

Early Neolithic around 8,000 - 7,000 BP [25] and in Makarovo 1 in layers dating to Late Neolithic layers [26]. The Cis-Baikal pottery has similar traits as the earliest pottery in North Asia, and is argued to have a south-east origin [27,28].

Angara river group (n=10)

The sites of Cyclodrome (Lokomotiv) (irk051) and Glazkovo (irk036) both belong to the Glazkovo necropolis in Irkutsk city. The nickname "*necropolis*" was given to Glazkovo due to the large number of cemeteries from different periods at the site [10,29]. The Glazkovo necropolis is situated in the city of Irkutsk, Sverdlovskiy Okrug. The first burials were accidentally discovered in 1887 and published by Vitkovskij in 1889, however there is some evidence that human remains were found at the area even earlier. Both Cyclodrome and Glazkovo were excavated by several researchers in the 20th century [30–32]. The Cyclodrome burial site is an Early Neolithic burial site with an abundance of red ochre in the burials [10]. This site belongs to the Kitoi culture, important features are presence of ochre and fishing hooks [10]. We studied one individual from the Locomotive site containing 98 burials, Irk051, which is dated to the late Mesolithic, i.e. the oldest phase of the cemetery [12] which was in use about 170 years in the chronology between 5140 ± 60 – 7140 ± 130 BCE [1].

The Glazkovo necropolis in Irkutsk contains burials belonging to both the Kitoi and the Glazkovo cultures [10]. The individual from Glazkovo (irk036) belongs to the Glazkovo culture, which dates to Late Neolithic - Early Bronze Age. The Glazkovo burials are characterized by the presence of white nephrite discs/rings, copper or bronze artefacts and pottery. The Suhaja Pad' Buret' site, burial 2 (irk025) is from the Early Bronze Age and consists of two burial groups. In at least one burial from both groups, Glazkovo culture artefacts have been found

such as white nephrite rings/discs and fragments, as well as traces, of metal objects (copper and bronze). Bigger nephrite tools, ivory and stone arrowheads, harpoons and fishing lures, fragments of personal ornaments - nacre beads and white cylinders - are also considered typical Glazkovo culture artefacts [10,13]. The Ust'-Dolgoe (Ust'-Dolgaya) site, burial 3 (irk022) is also a Glazkovo site, based on the burial inventory [10]. Chastaja (Chastye) Padi (irk033) is both a burial and a settlement site. The burials belong to Glazkovo culture while the settlement site had a layer with fragments of pottery with net-imprint [10]. Anosovo N1 (irk050) is an Eneolithic Glazkovo site, represented by two burials, both of which had the typical white nephrite discs in them [10]. One of the burials at Anosovo contained a needle with anthropomorphic decoration [13].

Podostrozhnoe is a term used for a group of sites, including Kirpichnyj Saraj (irk034), Gorodische site N 1 (irk040) and Podostrozhnoe site N 3 (irk057). In one of the burials from Kirpichnyj Saraj site fragments of pottery were found together with other artefacts, such as ivory bow fragments and arrowheads [10]. Some of the burials at Kirpichnyj Saraj and Gorodische site N 1 are classified as Serovo culture sites. The inventory of Serovo burials usually includes ivory bow fragments, spear heads and pottery. Podostrozhnoe site N 3 is a Glazkovo site. Burial 3 from the Zarubino site (irk032) is a Medieval burial.

Lena river group (n=9)

The valley of the Lena river is densely packed with ancient burial sites, with concentration of burials in Biryulka to Zhigalovo. The individuals analysed in the current study originate from seven sites. The archaeological sites are located in the valley of the upper basin of Lena river, situated in the central part of Baikal lake.

In the Lena river valley, a local Early Neolithic culture complex “Makrushynskaja” has been identified based on the findings from the Makrushino burial site. One important feature defining this cultural complex is the presence of stone structures above and in the burials. Another characteristic is the absence of fishing hooks of the “Kitoi” type and biface type arrowheads. The Late Neolithic group of this complex is considered “archaic” and the material culture bear similarities with the Serovo and the Isacovo traditions, but without ivory bow-parts. The burials often contain several individuals, lying straight on their backs and perpendicular to the river, with the feet towards it. Makrushino (burial 26, samples=mak026 and mak001) is a burial site containing material from the Early Neolithic to the Bronze Age. It is situated in the Kachug District, on a 8-10 metrewide terrace on the right bank of Lena river and was first excavated in 1989 [33]. After several years of excavations 24 distinct constructions had been recovered on the site [34]. Some of the stone-settings found indicate the presence of a grave but were found to be empty. The anthropological material from the site is rather well preserved and several radiocarbon dates were produced from the bones. The earliest absolute date is from burial 1 (7,340 ± 120 cal BP) and the latest from burial 3 (4,070 ± 900 cal BP) [35]. Burial 1 contained hooks and a fish-shaped lure, which are of Kitoi (culture)-type, and indicative of the cultural affiliation of the burial. Our dating of burial 26 corresponds to the Eneolithic period which is in agreement with the age of several other burials representing the Glazkovo culture at Makrushino [36], or the local Makrushyno culture.

The sites at Novyj Kachug (irk067) and Zvjozdochka (irk061) were both excavated by Okladnikov in 1930 at the left bank of Lena river. Both were classified as Glazkovo culture sites. Novyj Kachug is an example of how Glazkovo burials could be covered with flat rocks. They

were considered to be part of the “archaic group” [13]. At the right bank of Lena, the sites at Korkino (irk030), Shishkino N1 (irk068) and Makarovo (irk017) are also classified as Glazkovo culture sites. At Marakovo, the burials N1 and N2 have several typical white discs and nacre beads, as well as large fragments of pottery [13]. The Silinskij burial site (irk008) lies on the left bank of Lena River. On the opposite side of the river, 200 metres down the stream, the famous rock paintings at Shishkino cliffs are found (Shyshkinskie pisanicy). The Shishkino site is represented by three burials of the Glazkovo period, all with typical Glazkovo artefacts. Interestingly many ceramic fragments were included in the burials, which is uncommon [37].

Popovskij Lug (irk007) is a collective name used for a number of Neolithic-Bronze Age sites, all situated on the right bank of the Lena river in the Kachugsky District. The excavations started in 1948, and the location was first treated as a settlement site called “Bannyj Ruchej”. From the 1960s the site is called Popovskij Lug. During the excavations in 1972 a new burial site was found. It is situated in the area of the Popovskij Lug site (Upper Lena river, Irkutsk Oblast), on a 6-8 metrehigh terrace formation. Three burials were excavated, all of them close to the edge of the terrace. This burial site is of Glazkovo type, common in Cis-Baikal area during the Neolithic [38]. In 2010 an archaeological expedition led by Shergin from the Laboratory of Archaeology and Ethnography Pedagogical Institute, Irkutsk State University, excavated the site at Popovskij Lug II. They unearthed a single male burial (Irk007). The burial was found at a depth between 0.43 - 0.50 metres. The burial had an oval shape extending 1.08 x 1.0 metres, and was oriented diagonally southeast to northwest. Besides the osteological material there was an inventory of ivory, horn and stone, objects of “mobile art” and a fragment of “ware-pottery” of “Hajtinskij” type were found. Preliminarily, the burial was estimated to be of Early Neolithic Age. However, the first radiocarbon dating of this burial yielded radiocarbon age of

7,735 ± 69 cal BP (OX 27119). The burial, thus, is the oldest earliest documented burial in Cis-Baikal with ceramic as grave goods. The dating obtained in the present study was even slightly older (7,940 ± 30 BP, i.e. c.9,000 – 8,653 cal BP). Besides of the osteological material, Popovskij Lug is rich in ceramic fragments of “ware-pottery” (or *Schnurkeramik kultur*). The most interesting feature of Popovskij Lug is the amount of ceramic fragments from variety of pottery types found in deeper layers at the site [39].

Baikal lake (n=4)

The Mys Uyuga burial 2 (individual 1- irk071, individual 2-irk072) is a double burial, which is situated on the peninsula of Mys Uyuga, facing the Olkhon Island. It was excavated in 2016 [40] and found to contain two individuals, a male and female, holding hands. The burial has about 0.5 metres of depth and the individuals were placed on the base rock together with tools and personal ornaments. The burial is classified as belonging to the Glazkovo culture, of local Sagan-Nughe type (South Olkhon), which dates to the Bronze Age [41].

The Shamanka 2 site, complex 3 (sample = individual 1, irk076) is a site situated on the southern side of the cape Shamanka. It was excavated in 1998 [6]. The burial was covered by a stone construction, 0.8 by 2.3 metres in size. The individual was placed stretched out in the burial, with artefacts of bronze, tools and personal ornaments. The burial ritual is typical for the South Baikal territorial variant of the Glazkovo type, contemporaneous with the Glazkovo culture. The main sites of the Glazkovo period are located along the Angara and Lena rivers.

The Sokhter site, complex 1 contains a single burial (burial 5, irk075), discovered at the bank of the cove Sokhter, at the Muhorkskij bay of Baikal lake. It was excavated in 1994. The burial

construction included several layers of stone, covering the burial pit, which had a round shape and was 40 centimeters deep. The individual was buried in a “sat down position” with the knees bent to the body. No artefacts were discovered in connection to the burial [42]. This burial is a *Shumilikhinskaja* burial type, and dated to the Early Bronze Age [6].

Trans-Baikal group (n=7)

The analysed burials in the Trans-Baikal group are all located east of the Baikal lake. The earlier described observations in Cis-Baikal archaeology serves also as a frame for a similar regional archaeological periodization in the Eastern and Western Transbaikal regions (Zabaikalije) [43–45]. The sites are: Izvestkovaja-1 (burial 1-brn008 and burial 2-brn001), Nozhyj lake burial site-2 (burial 6, skeleton 3, brn012 and one individual from a burial without a number, brn002), Okoshki-1 (irk078), Dvorcy-Dacha burial (brn003) and Dzhylinda (irk00X). Limited information is available on the Stone Age of the area covering east of Baikal lake, some of the material is published in this paper for the first time. Chronologically the samples from Trans-Baikal are scattered between c.8,500 and 6,000 cal BP, with one considerably younger sample falling around 3,000 BP (Irk078) (**Fig. S3c)**. The Izvestkovaja-1 Neolithic burial site lies by the Kuenga river, 12 kilometers from the railway station of Verhnyaja Kuenga. The site was first excavated in 1999 [46]. The Nozhyj lake site-2 (n=2; brn002, brn012) is one of several archaeological sites located on the south-eastern side of Nozhyj lake, around 25 kilometers from Aginskoe. Excavations at the site-2 started in 1975 [47]. The site has layers from the Neolithic, Bronze Age and Iron Age. The radiocarbon dates obtained in the present study place the individuals in the Neolithic period. There are some elements of burial rite (red ochre, nephrite tools, arrowheads and blades), which allows us to treat Nozhyj burials as Kitoi culture burials.

Okoshki-1 is a Bronze Age site, located in Priargunsky District of Zabaykalsky Krai. We analysed the individual from Burial 23 [48]. The individual was placed in a burial pit under a layer of rocks, forming an oval-shaped stone cover measuring to ca 4.2 x 5.0 metres. The skeleton was positioned with bent knees, turned over to the right side. Dvorcy-Dacha contains a single burial located 20 km in the north-west direction of the Chita city Zabaykalsky Krai. It was accidentally discovered by I.I. Kirilov at the river Kadalinka terrace, by the Yablonovy Mountains, and remained unpublished. The radiocarbon dating of this individual allowed us to place the burial within the Neolithic context. The Dzhylinda site (also called Lower Dzhylinda site-1 at Sivakon) is an Early Neolithic site (sometimes mentioned as Mesolithic), located near in the valley of Vitim river, Zabaykalsky Krai. Vitim is a large water body, an eastern tributary to Lena river, covering the eastern side of Baikal lake [15]. The Dzhylinda site was discovered in 1977, the individual from this site was placed in a pit marked by stone slabs marking in, under a heavy rock setting. Besides the osteological material, the site provided a large inventory of Middle and Late Stone Age artefacts nephrite knives, beads, nucleuses, a fishing hook and other stone and ivory tools. The facial reconstruction of the individual allows to suggest a "mongoloid type" [49]. The skeleton bore marks or cannibalism [49].

Sakha Republic group (Yakutia) (n=9)

The samples from Yakutia cluster geographically in three areas: South-Western Yakutia (Khaiyrgas Cave at the Lena river (yak025)), central Yakutia (samples=N3a, N4a1, N4b2, N5a) and North-Eastern Yakutia (yak021-024). In Yakutia a periodisation was based on the material from multi-layered archaeological human activity sites, which allowed observations of changes in the form of ivory and stone tools and technological features during a period of a couple of thousands of years. This has led to a periodisation of the prehistory of the northeast

Asia that include cultural complexes which are present for a long time and today be considered general. The Yakutia group includes the following sites: Khaiyrgas cave (yak025), Onnyos (N5a), Kyordyughen (Kyordyughen 2, N4a1, Kyordyughen 1, burial 1, N4b2), Kamenka 2 (individual 1 - yak022, individual 2 - yak023 and individual 3 - yak024), Pomazkino (yak021), Dyupsya (N3a).

The Khaiyrgas cave (yak025) is a site with a complex stratigraphy located on the Middle Lena river [20,50]. The sample taken was a human deciduous molar fragment. The tooth was discovered in a Paleolithic horizon and carries characteristics which adhere to the east dental pattern with archaic traits [51]. The radiocarbon dating shows that the individual is from the Late Palaeolithic. The other samples from Yakutia are younger (**Fig. S3d)**.

The Onnyos burial (N5a) was discovered at the site with the same name in 1978. Onnyos is a Belkachi culture burial, based on the burials inventory and presence of red ochre. Radiocarbon dating for the sample is consistent with previous reports of radiocarbon dating of the same material [52]. The archaeological material of the site is from different periods, from the Mesolithic to the Bronze Age [53]. The skull of the individual was found 40 centimetres away from the rest of the skeleton. There was an arrow fragment in the left *coxae* of the individual. There were also two skeletons of a *mustelidae* and a *canidae* found in the burial. The *mustelidae* being placed between the tibia and the *canidae* by the feet of the individual. The individual was archaeologically classified as a male.

Kyordyughen is a Late Neolithic burial site [54,55]. There are two burials (burial 1 and 2) located at the site which contain the remains of three individuals. Kyordyughen 1 is a double burial,

where the first individual (1-1) (N4b2) was assessed as a male. The body was positioned in the grave in a stretched out position and covered with a shield, made from ivory plates. There was also a deconstructed armor placed in a bundle by his feet. It consists of ivory plates with perforations for assembling the pieces. Remains of a bow were found by the right side of the skeleton. The left *femur* was missing, and in its place there was as assemblage of bones from one more individual (1-2). There was also a number of artefacts found in the grave, including an adze and a concentration of arrow heads. The second burial (Kyordyughen 2) (N4a1) was represented by scattered bones on depth between 10 and 40 cm. Anthropological analysis of the individual N4b2 showed a combination of morphological traits found in Asia [56].

The Kamenka-II burial contained three juvenile individuals [52,57] (individual 1-yak022, individual 2-yak023 and individual 3-yak024). Individuals 1 and 2 were estimated to be between 6 and 9 years old, the individual 3 was estimated to be 2 months old. There are indications that the older children were buried in embellished clothing with several artefacts. There was a miniature flint knife by the remains of the youngest individual (presumably a girl by osteological criteria which was confirmed by the genetic analyses). A ceramic fragment was present in the burial. The measured nitrogen values, measured on deciduous teeth, for these individuals are high, which must be taken into consideration. The values are probably affected by weaning, see below. Anthropological studies showed that cranial and odontological features exhibited a mixture of both “arctic” and “north-eastern” traits [58].

Two burials were discovered at the site of Pomazkino-III in 1992 [52]. The genetic analyses were undertaken on a female individual from burial 2 (yak021). The molecular sex identification was in accordance with osteological assessment. There were several artefacts in the grave, two

crania of *mustelidae*, fragments of ceramics and a necklace and tools. Radiocarbon dating placed the material between Late Neolithic/ Early Bronze Age.

The Dyupsya burial (N3a) is a male burial dated to the Early Iron Age. The individual was positioned on the right side, with the legs close to the chest, in an "embryo" position with a stretched out back. The left leg from the knee down was absent. There are also gnawing marks on the left leg and the ribs, were presumably from carnivores. Additionally, there were marks of sharp weapon trauma on the left leg of the individual. A fragment of iron was found in the burial, marking the beginning of the Iron Age at this latitude [59]. The earliest period of Iron Age in Yakutia falls between 8th -5th century BC [60].

Krasnoyarsk Krai and Amur Oblast (n=2)

Krasnoyarsk Krai is represented by Nefteprovod-2 site, burial 1 (kra001). The burial was discovered by the archaeological expedition of Siberian Federal University. Blagoveschensky District of Amur Oblast is represented by Oktyabr'skoe site, burial 2 (sample = bla001).

Radiocarbon dating and stable isotope analysis

Calibration of the conventional radiocarbon ages were performed using Oxcal version 4.3.2 [61], and the IntCal13 atmospheric curve [62]. Calibrated results were reported with 95% confidence intervals (2$^{\sigma}$). Isotopic variation and, thus, the source of dietary, may affect the integrity of the dates due to reservoir effects. IRMS stable isotope analyses were performed on the same samples that were radiocarbon dated. The analyses were done at Beta Analytic Inc. Earlier analyses of stable isotopes, have shown that the inclusion of fresh water fish was common but of different importance in different areas of Siberia [16,63–65].

The isotopic values obtained here confirm that there was a regional variation in dietary patterns in accordance with earlier analyses. The isotopic values for a few samples from Yakutia and Cis-Baikal, exhibit elevated $\delta^{15}N$-values (>+16). A few of these values were measured on deciduous teeth and probably exhibit an effect of weaning. However, not all of the teeth with elevated values are deciduous and probably they exhibit an effect of fresh water fish consumption (**Fig. S3e-g)**. Thus, the consumption of fish probably causing a freshwater reservoir effect (FRE) to some extent also may affect some of the age estimations. This does not have major consequences for the present study.

REFERENCES

1. Bazaliiskii, V. I. Burial complexes of the epoch of Late Mesolithic- Neolithic of Baikal Siberia: burial rites, absolute age. *Rep. Lab. Anc. Technol. Irkutsk Natl. Res. Tech. Univ.* **Number 9,** 43–101 (2012).
2. Konstantinov, M. V. Cultural-historical etaps of ancient history of south-west of Transbaikalia. in *Archaeological quest (North Asia).* (ed. Medvedev, V. E.) 16–24 (Nauka., 1980).
3. Konstantinov, M. V. and K., A. V. Historical heritage of archaeological discoveries and epochs. *Small Encicl. Transbaikalia Archaoeology Part 1* 12–40 (2011).
4. Losey, R. J., Waters-Rist, A. L. .. Nomokonova, T. .. and Kharinskii, A. . A second mortuary hiatus on Lake Baikal in Siberia and arrival of small-scale pastoralism. *Sci. Rep.* **7,** (2017).

5. Mochanov, Y. A. & Bland, R. L. The Most Ancient Paleolithic of the Diring and the Problem of a Nontropical Origin for Humanity. *Arct. Anthropol.* **30,** 22–53 (1993).
6. Turkin, G. V. and K., A. V. Burial site Shamanka II: the question of chronological and cultural affinities of the burial complexes from the Neoltihic-Bronze Age at the South Baikal. *Bull. Irkutsk State Univ.* 124–158 (2004).
7. Weber, A. W. *et al.* Hunter–gatherer foraging ranges, migrations, and travel in the middle Holocene Baikal region of Siberia: Insights from carbon and nitrogen stable isotope signatures. *J. Anthropol. Archaeol.* **30,** 523–548 (2011).
8. Weber, A. W., Link, D. W. and Katzenberg, M. . Hunter-gatherer culture change and continuity in the Middle Holocene of the Cis-Baikal, Siberia. *J. Anthropol. Archaeol.* **21,** 230–99 (2002).
9. Weber, A. W., Beukins, R. .. Bazaliiskii, V. I. .. Goriunova, O. I. .. Savel'ev, N. A. Radiocarbon dates from Neolithic and Bronze Age hunter-gatherer cemeteries in the Cis-Baikal region of Siberia. *Radiocarbon* **48,** 1–40 (2006).
10. Okladnikov, A. P. *Neolithic and Bronze Ages of Cis-Baikal. Part I & II.* (Academy of Scineces USSR, 1950).
11. Mamonova, N. N. and S., L. D. Radiocarbon chronology of Holocene burials of Cis-Baikal and Transbaikal using osteological material from the cemeteries. in *People, adaptation, culture* (ed. Sorokin, A. N.) 127–138 (Institute of Archaeology of the Russian Academy of Sciences, 2008).
12. Bazaliiskii, V. I. and S., N. A. The Wolf of Baikal: the "Lokomotiv" Early Neolithic Cemetery in Siberia (Russia). *Antiquity* **77,** 20–30 (2003).
13. Okladnikov, A. P. *Neolithic and Bronze Ages of Cis-Baikal. Part III.* (Academy of Scineces USSR, 1955).
14. Okladnikov, A. P. *Neolithic sites of Angara river.* (Nauka, 1974).

15. Vetrov, V. M., Zadonin, O. V. and Ineshin, E. M. Multi-layered site of Lowed Dzhylinda (Sivakon)-1 in Bambujskaya hollow. in *Cultures and sites of the Stone Age and age of Early Metal of Transbaikalia.* 98–112 (Nauka, 1993).
16. Weber, A. W., White, D. .. Bazaliiskii, V. I. .. Goriunova, O. I. .. Savel'ev, N. A. and Katzenberg, M. A. Hunter–gatherer foraging ranges, migrations, and travel in the middle Holocene Baikal region of Siberia: Insights from carbon and nitrogen stable isotope signatures. *J. Anthropol. Archaeol.* **30,** 523–548 (2011).
17. Mamonova, N. N. & S., L. D. Experience of dating with 14C of the Cis-Baikal burials. in *Soviet Archaeology* **1,** 19–31 (Nauka, 1989).
18. Bazaliiskii, V. I. Mesolithic and Neolithic mortuary complexes in the Baikal region of Siberia. in *Prehistoric Hunter-Gatherers of the Baikal Region, Siberia: Bioarchaeological Studies of Past Life Ways.* (ed. Weber, A. W., Katzenberg, M. A. .. and Schurr, T. G.) 51–86 (University of Pennsylvania Museum Press, 2011).
19. Weber, A. W. and B. R. Middle Holocene hunter-gatherers of Cis-Baikal, Siberia: An overview for the new century. *J. Anthropol. Archaeol.* **29,** 491-506. (2010).
20. Kuzmin, Y. V. The origins of pottery in East Asia and neighbouring regions: An analysis based on radiocarbon data. *Quat. Int.* **441,** 29–35 (2017).
21. Kuzmin, Y. V. and O., L. A. The Neolithization of Siberia and the Russian Far East: radiocarbon evidence. *Antiquity* **74,** 356–364 (2000).
22. Boaretto, E., Wu, X. .. Yuan, J. .. Bar-Yosef, O. .. Chu, V. .. Pan, Y. .. Liu, K. .. Cohen, D. .. Jiao, T. .. Li, S. .. Gu, H. .. Goldberg, P. and Weiner, S. Radiocarbon dating of charcoal and bone collagen associated with early pottery at Uychanyan Cave, Hunan Province, China. *Proc. Natl. Acad. Sci.* **106,** 9595–9600 (2009).

23. Xiaohong Wu, C. Z., Paul Goldberg, David Cohen, Yan Pan, Trina Arpin and Ofer Bar-Yosef. China-Early pottery at 20 000 years ago in Xianrendong Cave, China. *Science* **336,** 1696–1700 (2012).
24. Kuzmin, Y. V. *Geochronology and paleoenvironment of Late Palaeolithics and Neolithics of moderate belt of East Asia*. (Pacific Geographical Institute, 2005).
25. Shergin, D. L. Ceramics of Popovskij Lug site at Upper Lena river. (Based on the materials of archaeological field work of 2003). in *Origins, formation and development of the Eurasian poly-culturalism. Cultures and societies of the North Asia in historical past and modern times. Materials of XLV Russian, with international participation, archaeological and ethnographical students and y* 173–174 (Irkutsk, 2005).
26. Vetrov, V. M. Upper Makarovo I complex. in *Ancient history of the people of the south of East Siberia.* (ed. Vetrov, V. M. and Z., V. S.) 146–159 (Irkutsk, 1974).
27. Vetrov, V. M. Problem of the similarities of the technology of the production and ornamenting the pottery from the early ceramic complexes of North Asia. in *Modern problematics of archaeology in Russia* 173–176 (Insittue of Archaoelgy and Ethnograph. SBRAS., 2006).
28. Vetrov, V. M. The most ancient traces of ceramics production on the East Asia (problems of the emergence, periodization, terminology). in *Anthropocene. Paleoanthropology, geoarchelogy, ethnology of Asia.* 29-34. (Ottisk, 2008).
29. Okladnikov, A. P. The Peoples of Siberia. in *Ancient population of Siberia and its cultures* (eds. Levin, M. & Potapov, L.) 13–98 (University of Chicago Press, 1964).
30. Bazaliiskii, V. I., Menshagin, E. V. .. Golbert, V. V. and Prokopijev, V. N. New data about Kitoi culture. in *Problems of archaeology and ethnography of Siberia* 68–69 (Irkutsk State University, 1982).

31. Khoroshikh, P. P. Neolithic cemetery at the “Lokomotiv” stadium in Irkutsk. in *Siberian archaeological miscellany.* (ed. Okladnikov, A. P.) 84–93 (Novosibirsk. Nauka., 1966).
32. Ovchinnikov, M. P. Materials for the study of the ancient objects in the surroundings of Irkutsk. *Proc. Imp. Russ. Geogr. Soc.* **XXXV,** 62–76 (1904).
33. Vetrov, V. M., Berdnikova, N. E. .. Zhuravljov, A. I. and Frolov, A. V. Makrushyno site: culture and chronology. in *Paleoethnology of Siberia* 137–139 (Irkutsk, 1990).
34. Altuhov, V. V. Investigation of Makrushynskij burial site at the Upper Lena river. in *Second far-eastern conference of young historians: Thesis Presentation.* 42–43 (Far Eastern Branch of the Russian Academy of Sciences, 1992).
35. Vetrov, V. M., Berdnikova, N. E. .. Altuhov, V. V. and Frolov, A. V. Makrushyno site. Early Neolithic complex. in *Baikal Siberia in the ancinet times.* 112–132 (Irkutsk State Univeristy, 1995).
36. Altuhov, V. V. Makrushynskij burial site. Early Bronze Age complex. in *At the verge of generations: Materials of the Regional archaeological-ethnographical conference of students.* 100–100 (Center for children and youth tourism and local history, 2001).
37. Belonenko, V. V. and M., E. V. Silinskij burial at Upper Lena river. in *Ancient Baikal Siberia: miscellany of scientific papers* 51–65 (Irkutsk State Pedagogical College, 2000).
38. Aksenov, M. P. Survey at Lena river. *Archaeol. Discov. 1972 Nauka* 196–197 (1973).
39. Shergin, D. L. On the history of research of the site of Popovskij Lug at the Upper Lena river. in *Ancient cultures of Mongolia and Baikal Siberia: material from international scientific conference.* 56–60 (Buryat State University, 2010).
40. Kichigin, D. E., Emel’janova, Y. A. and Korostelev, A. M. An early Bronze Age double burial from burial site of Mys Uyuga. in *Ancient Cultures of Mongolia, Baikal Siberia and Northern China* (2017).

41. Novikov, A. G. and G., O. I. Burial practice of hunter-fishers from the Bronze Age of Pre-Olkhonie. in *Ancinet cultures of Mongolia and Baikal Siberia. Part 2* 81–85 (Tuvan State University, 2014).

42. Sosnovkaja, N. S. and K., A. V. Burial at the bank of the bay Sokhter and some aspects of studying of the “sitted” burials of Cis-Baikal. in *Dulovskij readings 1997, section of archaeology and ethnography: materials of seminar and announcement.* 70–73 (Irkutsk, 1997).

43. Ivashyna, L. G. Research in Buryatia. *Archaeol. Discov. 1977 Nauka* 232–233 (1978).

44. Kirillov, I. I. and K., A. K. Archaeology in Transbaikalia: problems and perspectives. in *Chronological stratigraphy of Palaeolithic of North, Central and East Asia and America* 194–198 (Academy of Scineces USSR, 1990).

45. Konstantinov, M. V. *The stone age of the eastern part of Baikal Asia*. (SB RAS, 1994).

46. Nesterenko, V. V. Neolithic burials in the valley of Shilka river. Culture and history of ancient and modern societies of Siberia and Far East. in *Materials of XLII Regional archaeological and ethnographical student conference.* 175–176 (Omsk State Pedagogical University, 2002).

47. Okladnikov, A. P. and K., I. I. *South-East Transbaikalia in the Stone Age and Early Bronze Age*. (Nauka, 1980).

48. Kharinskii, A. V., Kovychev, E. V. and Kradin, N. N. Burial site Okoshki I in south-east Transbaikalia and some aspects of identity of Mongols in XIII—XIV century. in *Dialogue of urban and steppe cultures in the Eurasian range. Historical geography of Golden Horde: materials of the seventh international conference, dedicated to the memory of Fjodorov-Davydov G.A.* (ed. Bocharov, S. G. and S., A. G.) 65–68 (Stratum Plus, 2016).

49. Peshemskij, D. V. and R., G. V. Human from Lower Dzhylinda I (preliminary study). *Anthropol. Bull. Sci. Alm. RAS* **No 4,** 179–180 (1998).

50. Stepanov, A. D., Kirillin, A. S., Vorob’ev, S. A., Solov’eva, E. N. & Efimov, N. N. Khaiyrgas cave at the Middle Lena (results of research of 1998 – 1999). in *Ancient cultures of North-Eastern Asia. Astroarchaeology. Paleoinformatics.* 98–113 (Nauka, 2003).

51. Zubova, A. V., Stepanov, A. D. and Kuzmin, Ya. V. Comparative analysis of a Stone Age human tooth fragment from Khaiyrgas Cave on the Middle Lena (Yakutia, Russian Federation). *Anthropol. Sci.* **124,** 135–143 (2016).

52. Mochanov, Y. A. & Fedoseeva S.A. *Sketches of prehistory of Yakutia. Stone Age: in 2 parts. Part 2 - Yakutsk*. 489–489 (2013).

53. Mochanov, Y. A. *et al. Archaeological sites of Yakutia*. (Nauka, 1983).

54. Alekseyev, A. N., Zhirkov, E. K., Stepanov, A. D., Sharaborin, A. . & Alekseeva, L. . Burial of an Ymyiakhtakh Warrior in Kyordyughen, Yakutia. *Archaeol. Ethnol. Anthropol. Eurasia* **26 (2),** 45–52 (2006).

55. Stepanov, A. D., Kuzmin, Ya. V. .. Hodgins, G. W. L. .. Jall, A. J. T. Kyordyugen site, Ymyiakhtakh culture, Yakutia: An interpretation of burial rite. *Archaeol. Ethnol. Anthropol. Eurasia* **N 40,** 51–61 (2012).

56. Chikisheva, T. A. & Pozdniakov, D. V. Anthropological investigation of the Ymyiakhtakh warrior in Kyordyughen. in *Problems of archaeology and ethnography of Siberia and adjacent territories. Materials of the Annual Session of the Institute of Archaeology and Ethnography of SB RAS, 2006* 234–240 (Institute of Archaeology and Ethnography of SB RAS, 2006).

57. Kashin, V. A. Neolithic burial of children at Middle Kolyma river. *Archaeol. Ethnol. Anthropol. Eurasia* **2,** 78–81 (2001).

58. Shpakova, E. G. Anthropological characteristics of the burial of a child from Late Neolithic Age from the site of Kamenka II. *Archaeol. Ethnogr. Anthropol. Eurasia* **2,** 140–153 (2001).
59. Stepanov, A. D. Early Iron Age Dyupsya burial, Central Yakutia. *Elsevier* **38,** 32–32 (2010).
60. Stepanov, A. D., Kuzmin, Y. V. and Jall, E. J. T. New data on the chronology of the Early Iron Age of Yakutia. *Bull. Irkutsk State Univ. Ser. "Geoarchaeology Ethnol. Anthropol.* 106–112 (2014).
61. Bronk Ramsey, C. Bayesian analysis of radiocarbon dates. *Radiocarbon* **Vol 51,** 337–360 (2009).
62. Reimer, P. J. *et al.* IntCal13 and Marine13 radiocarbon age calibration curves 0-50,000 years cal BP. *Radiocarbon* **55,** 1869–1887 (2013).
63. Katzenberg, M. A., Bazaliiskii, V. I. .. Goriunova, O. I. .. Savel'ev, N. A. .. and Weber, A. W. Diet Reconstruction of Prehistoric Hunter-Gatherers in the lake Baikal Region. in *Prehistoric Hunter-Gatherers of the Baikal Region, Siberia: Bioarchaeological Studies of Past Life Ways* (ed. Weber, A. W., Katzenberg, M. A. .. and Schurret. T. .) 175–191 (University of Pennsylvania Press, Inc., 2009).
64. Losey, R. S., Katzenberg, M. A. .. and Nomokonova, T. Middle Holocene Fishing and Hunting in the Baikal Region of Siberia. in *The Oxford Handbook of the Archaeology of Diet.* (ed. Lee-Thorp, J. and K., M. .) (Oxford University Press, 2015). doi:10.1093/oxfordhb/9780199694013.013.21
65. Mooder, K. P., Thomson, T. A., Weber, A. W., Bazaliiskii, V. I. & Bamforth, F. J. Uncovering the genetic landscape of prehistoric Cis-Baikal. in *Prehistoric hunter-gatherers of the Baikal Region, Siberia: Bioarchaeological Studies of Past Life Ways* (ed. Weber, A. W.) 107–120 (University of Pennsylvania Press, 2009).

Supplementary Figures

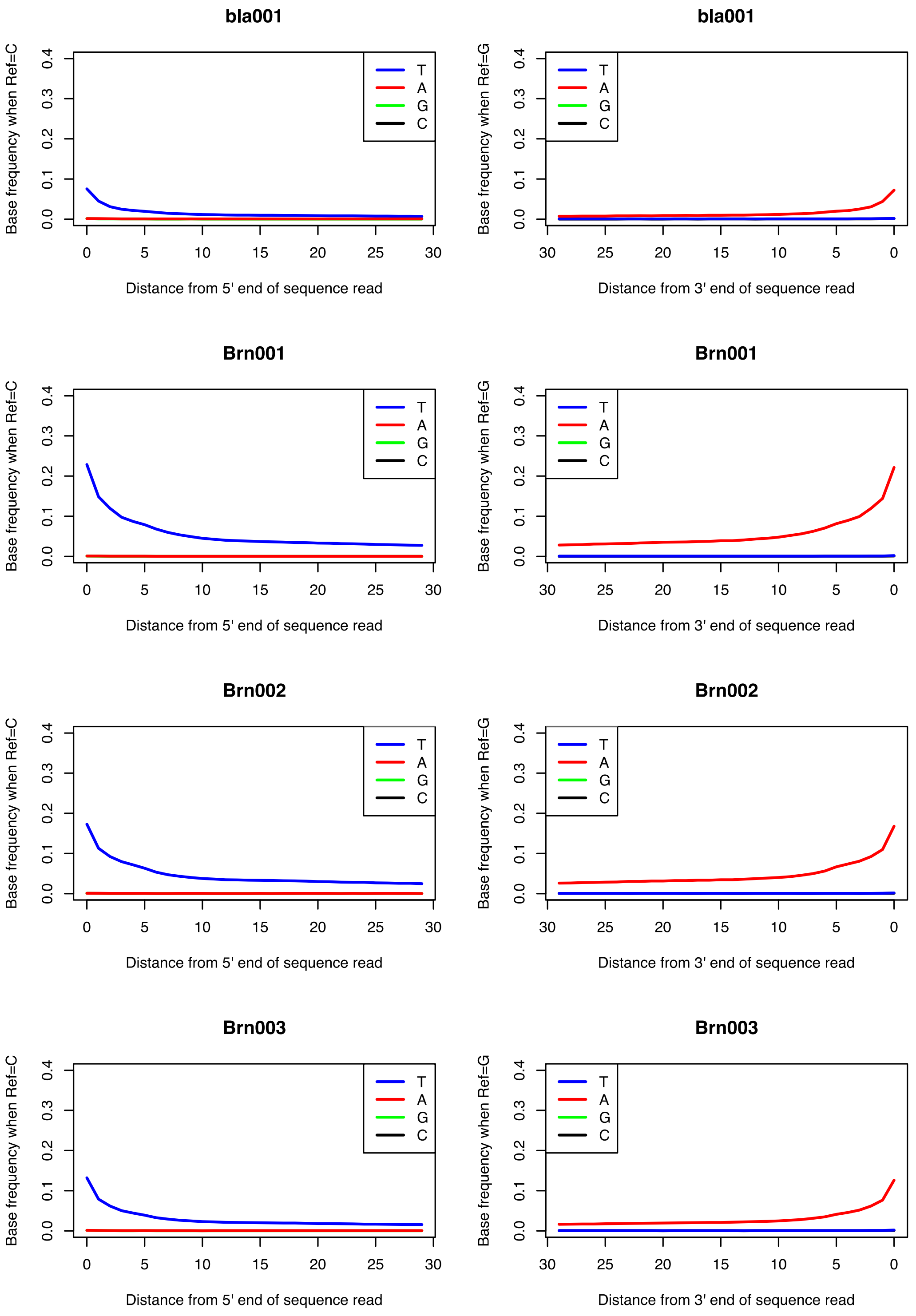

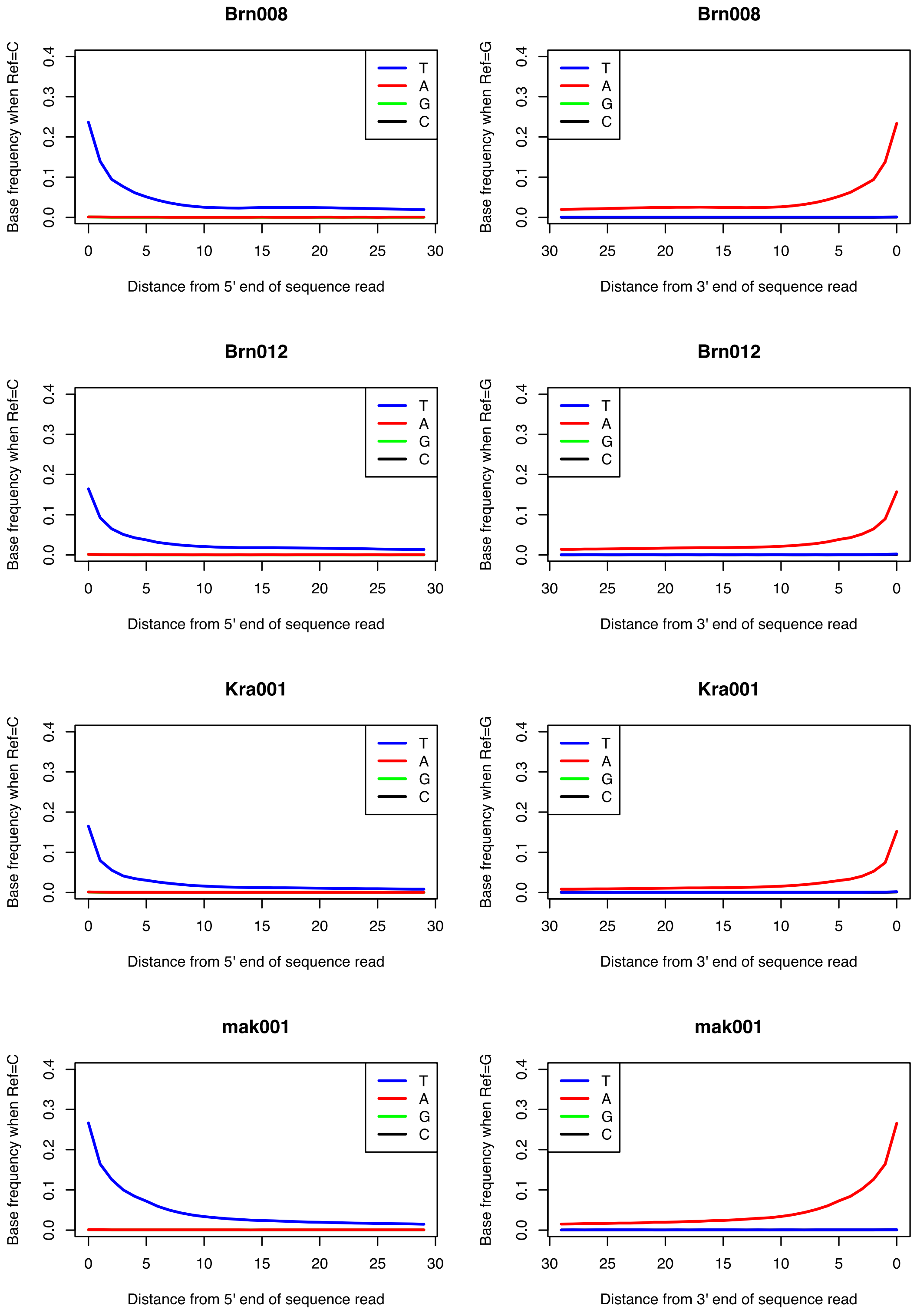
Brn008
Base frequency when Ref=C
Distance from 5' end of sequence read
T
A
G
C
Brn008
Base frequency when Ref=G
Distance from 3' end of sequence read
T
A
G
C
Brn012
Base frequency when Ref=C
Distance from 5' end of sequence read
Brn012
Base frequency when Ref=G
Distance from 3' end of sequence read
Kra001
Base frequency when Ref=C
Distance from 5' end of sequence read
Kra001
Base frequency when Ref=G
Distance from 3' end of sequence read
mak001
Base frequency when Ref=C
Distance from 5' end of sequence read
mak001
Base frequency when Ref=G
Distance from 3' end of sequence read

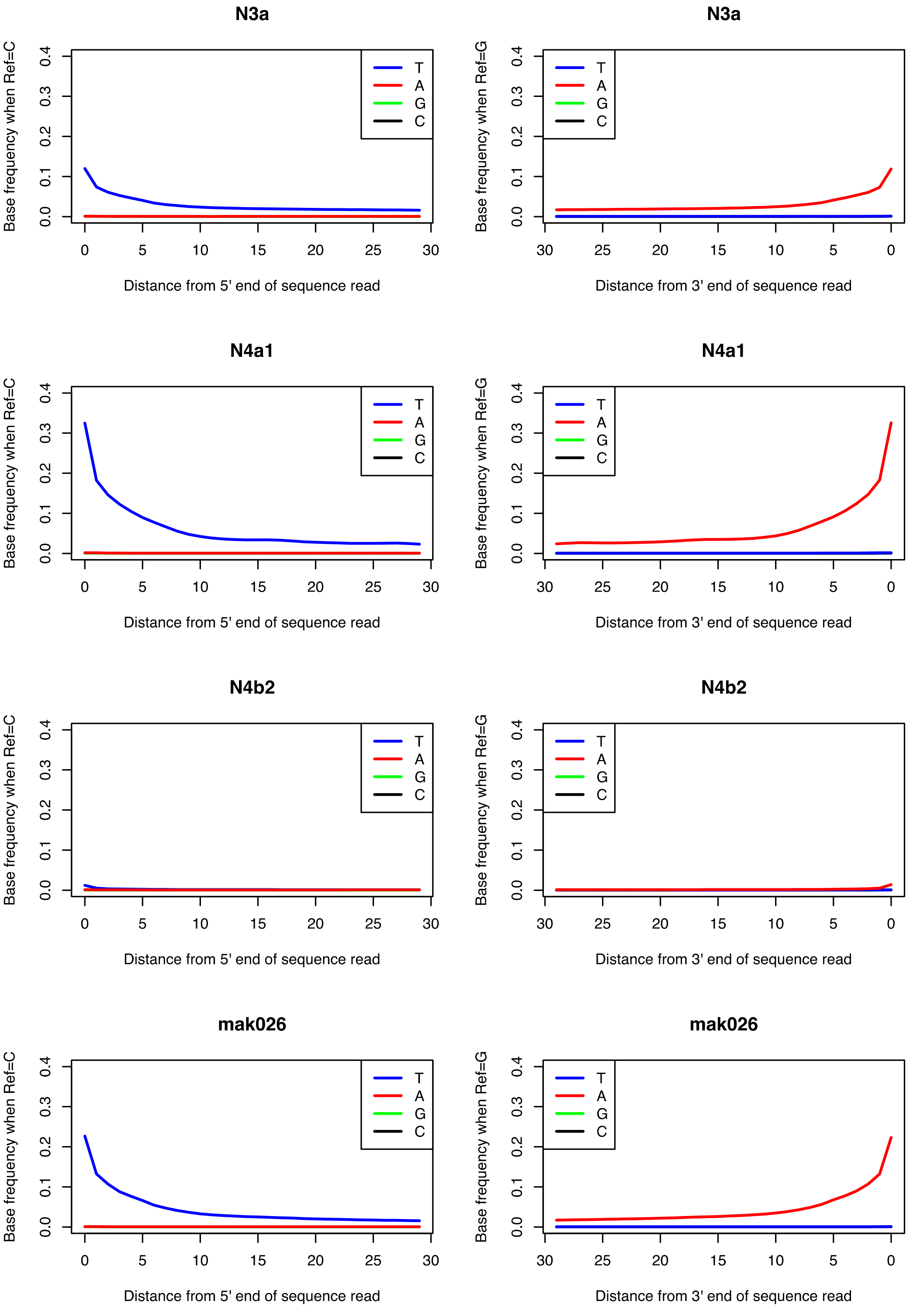
N3a
Base frequency when Ref=C
0.0 0.1 0.2 0.3 0.4
T A G C
0 5 10 15 20 25 30
Distance from 5' end of sequence read
N3a
Base frequency when Ref=G
0.0 0.1 0.2 0.3 0.4
T A G C
30 25 20 15 10 5 0
Distance from 3' end of sequence read
N4a1
Base frequency when Ref=C
0.0 0.1 0.2 0.3 0.4
T A G C
0 5 10 15 20 25 30
Distance from 5' end of sequence read
N4a1
Base frequency when Ref=G
0.0 0.1 0.2 0.3 0.4
T A G C
30 25 20 15 10 5 0
Distance from 3' end of sequence read
N4b2
Base frequency when Ref=C
0.0 0.1 0.2 0.3 0.4
T A G C
0 5 10 15 20 25 30
Distance from 5' end of sequence read
N4b2
Base frequency when Ref=G
0.0 0.1 0.2 0.3 0.4
T A G C
30 25 20 15 10 5 0
Distance from 3' end of sequence read
mak026
Base frequency when Ref=C
0.0 0.1 0.2 0.3 0.4
T A G C
0 5 10 15 20 25 30
Distance from 5' end of sequence read
mak026
Base frequency when Ref=G
0.0 0.1 0.2 0.3 0.4
T A G C
30 25 20 15 10 5 0
Distance from 3' end of sequence read

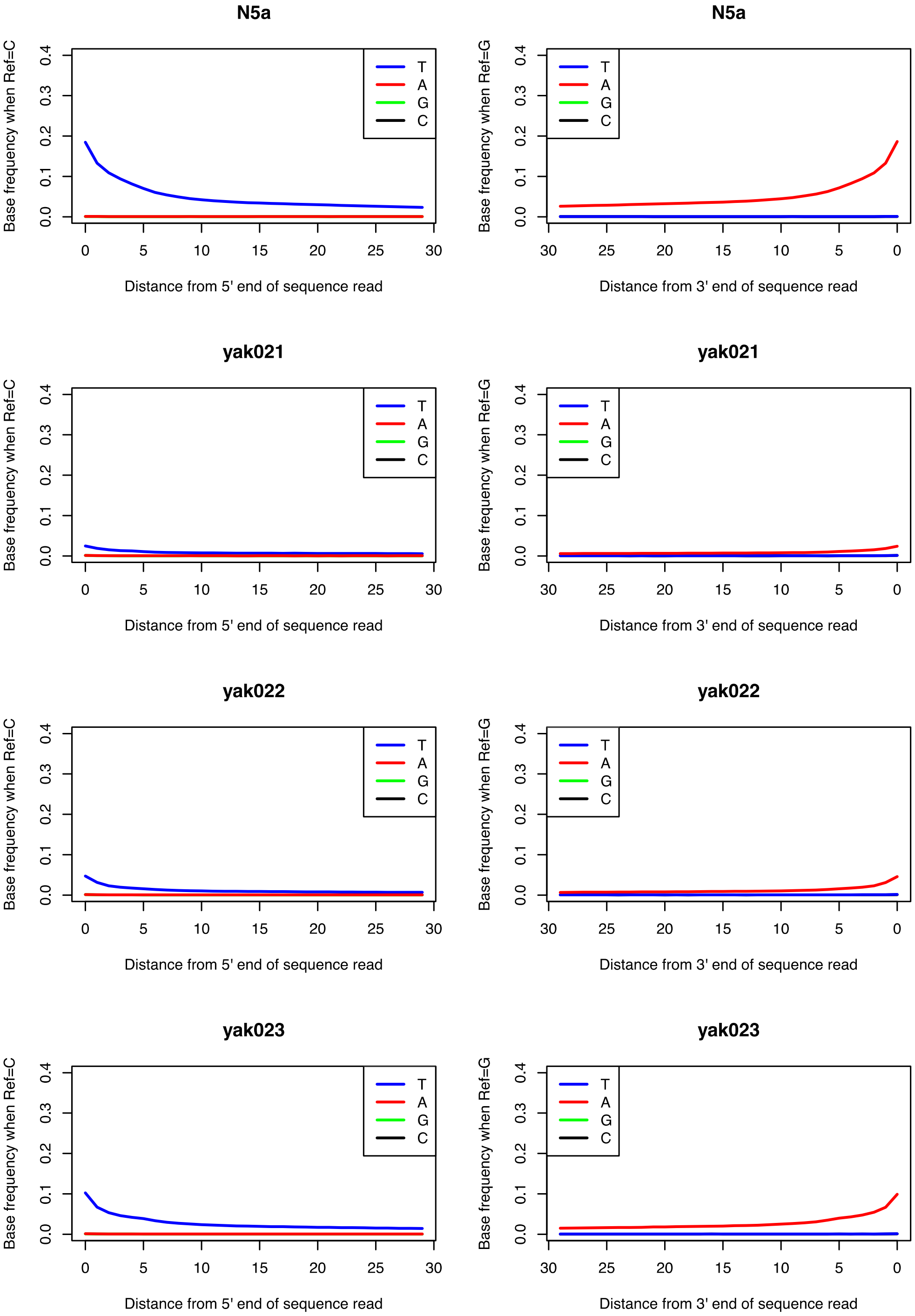
N5a
N5a
yak021
yak021
yak022
yak022
yak023
yak023
Base frequency when Ref=C
Base frequency when Ref=G
0.0 0.1 0.2 0.3 0.4
T
A
G
C
0 5 10 15 20 25 30
30 25 20 15 10 5 0
Distance from 5' end of sequence read
Distance from 3' end of sequence read

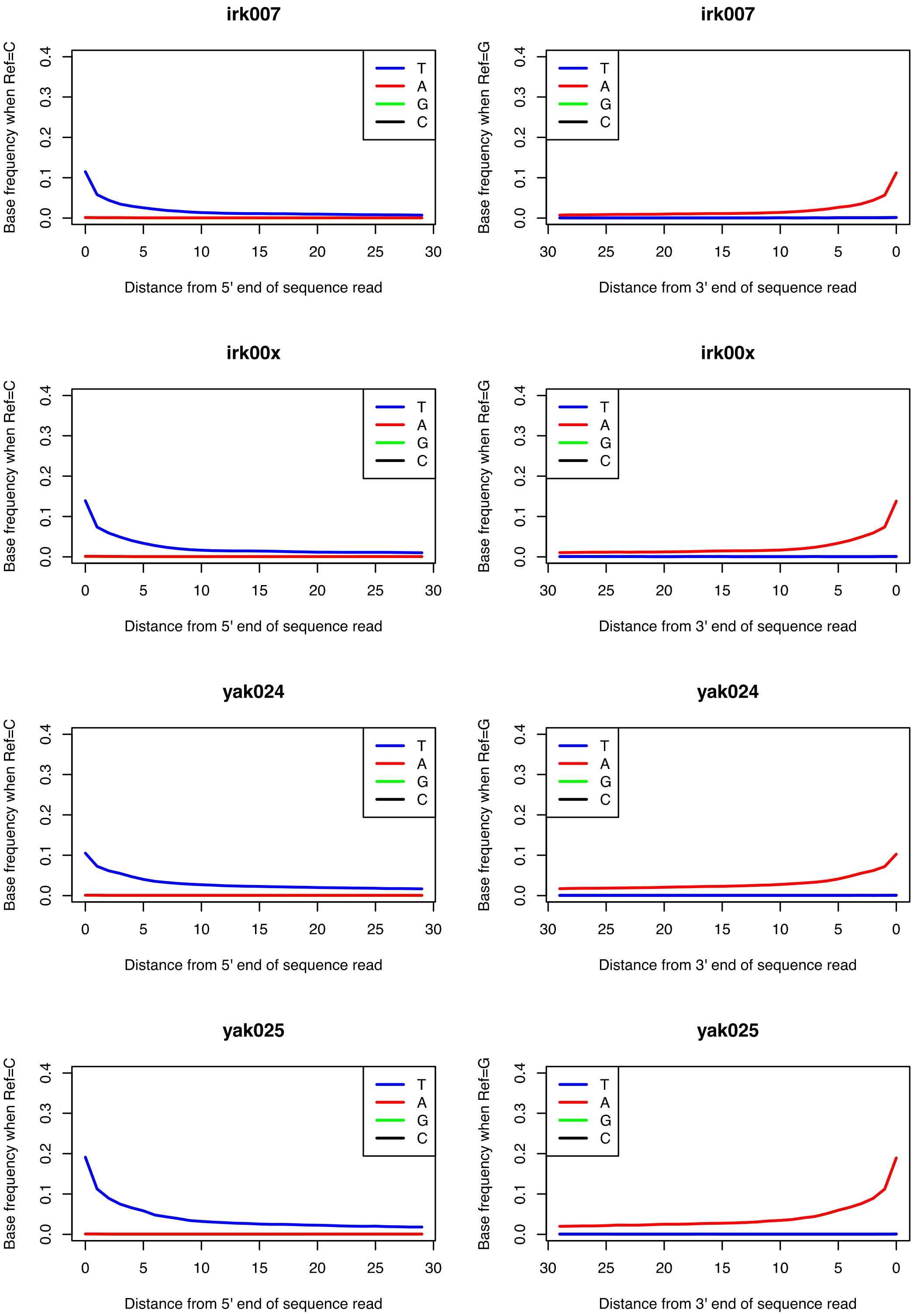

irk007
Base frequency when Ref=C
0.0
0.1
0.2
0.3
0.4
T
A
G
C
0
5
10
15
20
25
30
Distance from 5' end of sequence read
irk007
Base frequency when Ref=G
0.0
0.1
0.2
0.3
0.4
T
A
G
C
30
25
20
15
10
5
0
Distance from 3' end of sequence read
irk00x
Base frequency when Ref=C
0.0
0.1
0.2
0.3
0.4
T
A
G
C
0
5
10
15
20
25
30
Distance from 5' end of sequence read
irk00x
Base frequency when Ref=G
0.0
0.1
0.2
0.3
0.4
T
A
G
C
30
25
20
15
10
5
0
Distance from 3' end of sequence read
yak024
Base frequency when Ref=C
0.0
0.1
0.2
0.3
0.4
T
A
G
C
0
5
10
15
20
25
30
Distance from 5' end of sequence read
yak024
Base frequency when Ref=G
0.0
0.1
0.2
0.3
0.4
T
A
G
C
30
25
20
15
10
5
0
Distance from 3' end of sequence read
yak025
Base frequency when Ref=C
0.0
0.1
0.2
0.3
0.4
T
A
G
C
0
5
10
15
20
25
30
Distance from 5' end of sequence read
yak025
Base frequency when Ref=G
0.0
0.1
0.2
0.3
0.4
T
A
G
C
30
25
20
15
10
5
0
Distance from 3' end of sequence read

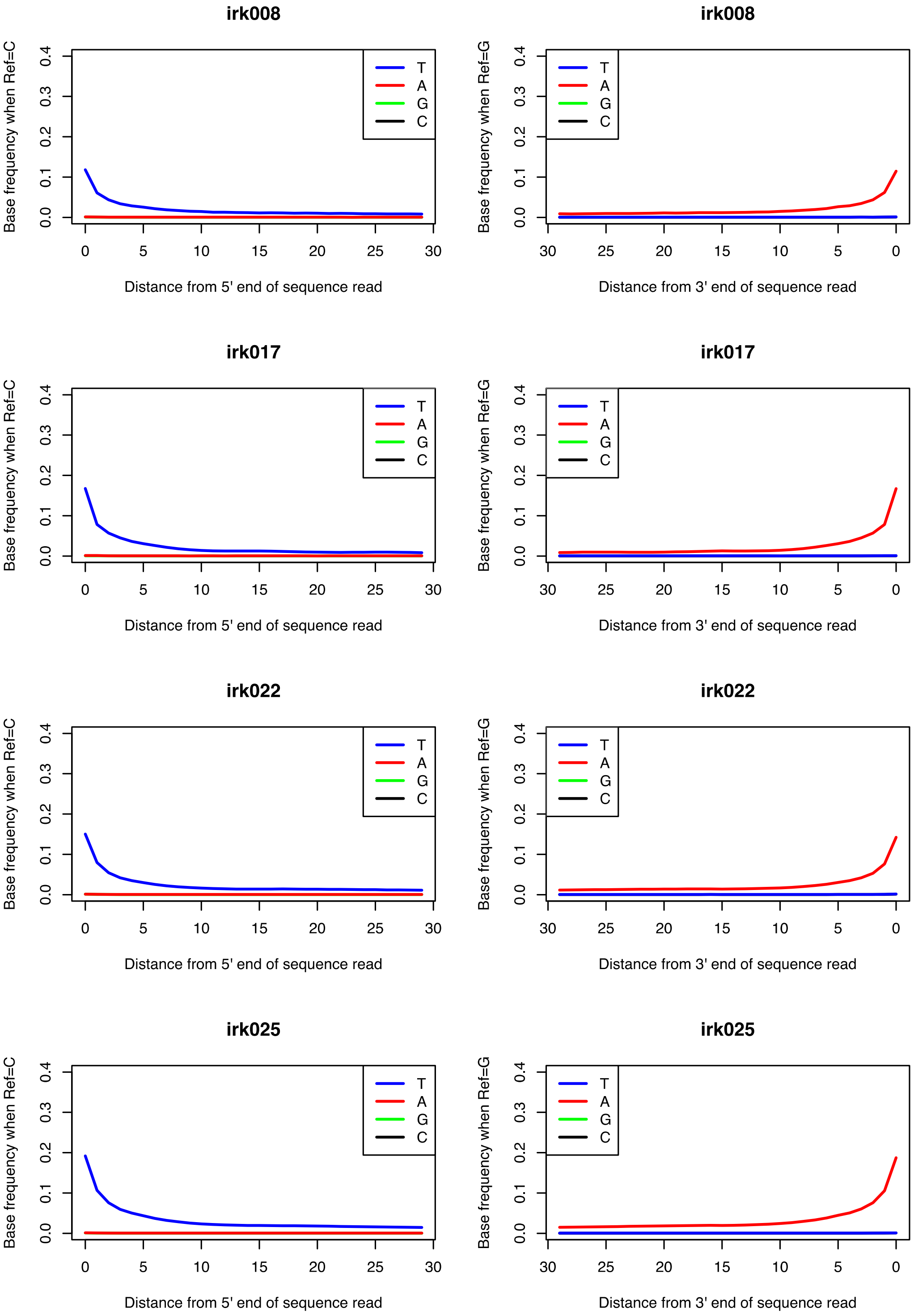
irk008
Base frequency when Ref=C
0.0 0.1 0.2 0.3 0.4
T A G C
0 5 10 15 20 25 30
Distance from 5' end of sequence read
irk008
Base frequency when Ref=G
0.0 0.1 0.2 0.3 0.4
T A G C
30 25 20 15 10 5 0
Distance from 3' end of sequence read
irk017
Base frequency when Ref=C
0.0 0.1 0.2 0.3 0.4
T A G C
0 5 10 15 20 25 30
Distance from 5' end of sequence read
irk017
Base frequency when Ref=G
0.0 0.1 0.2 0.3 0.4
T A G C
30 25 20 15 10 5 0
Distance from 3' end of sequence read
irk022
Base frequency when Ref=C
0.0 0.1 0.2 0.3 0.4
T A G C
0 5 10 15 20 25 30
Distance from 5' end of sequence read
irk022
Base frequency when Ref=G
0.0 0.1 0.2 0.3 0.4
T A G C
30 25 20 15 10 5 0
Distance from 3' end of sequence read
irk025
Base frequency when Ref=C
0.0 0.1 0.2 0.3 0.4
T A G C
0 5 10 15 20 25 30
Distance from 5' end of sequence read
irk025
Base frequency when Ref=G
0.0 0.1 0.2 0.3 0.4
T A G C
30 25 20 15 10 5 0
Distance from 3' end of sequence read

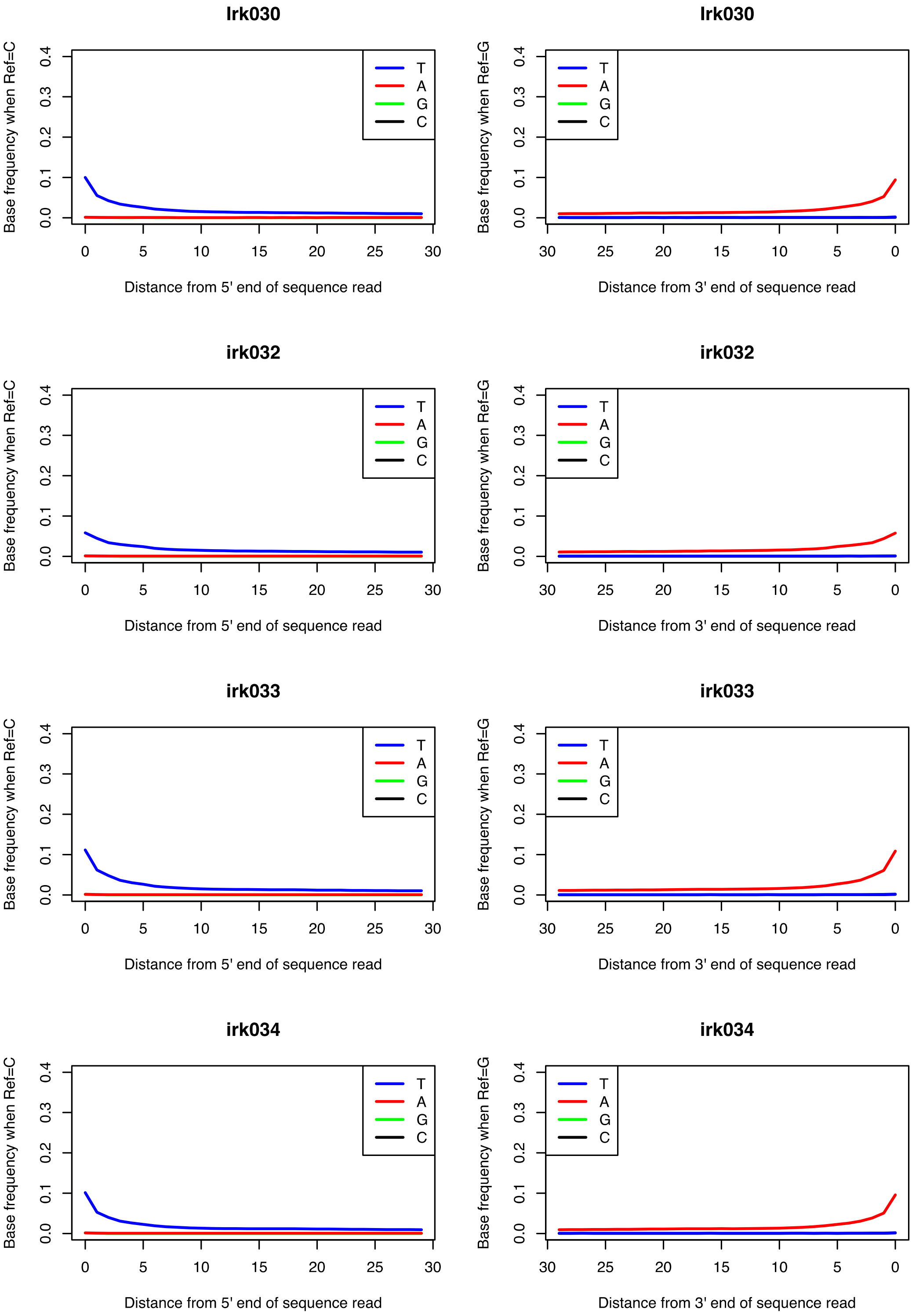
Irk030
Base frequency when Ref=C
Distance from 5' end of sequence read
T
A
G
C
Irk030
Base frequency when Ref=G
Distance from 3' end of sequence read
T
A
G
C
irk032
Base frequency when Ref=C
Distance from 5' end of sequence read
T
A
G
C
irk032
Base frequency when Ref=G
Distance from 3' end of sequence read
T
A
G
C
irk033
Base frequency when Ref=C
Distance from 5' end of sequence read
T
A
G
C
irk033
Base frequency when Ref=G
Distance from 3' end of sequence read
T
A
G
C
irk034
Base frequency when Ref=C
Distance from 5' end of sequence read
T
A
G
C
irk034
Base frequency when Ref=G
Distance from 3' end of sequence read
T
A
G
C

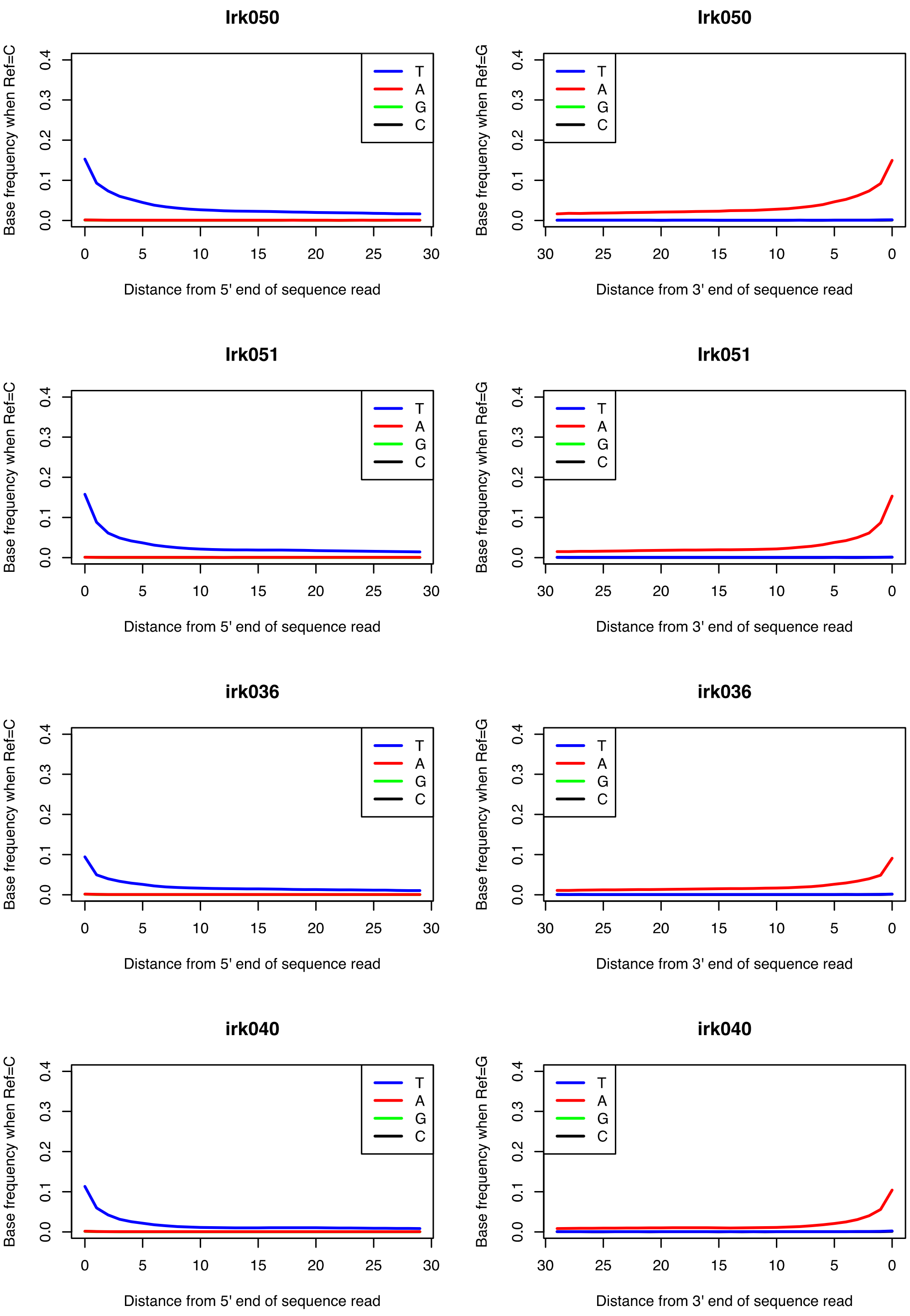

lrk050
lrk050
Base frequency when Ref=C
Base frequency when Ref=G
T
A
G
C
Distance from 5' end of sequence read
Distance from 3' end of sequence read
lrk051
lrk051
irk036
irk036
irk040
irk040

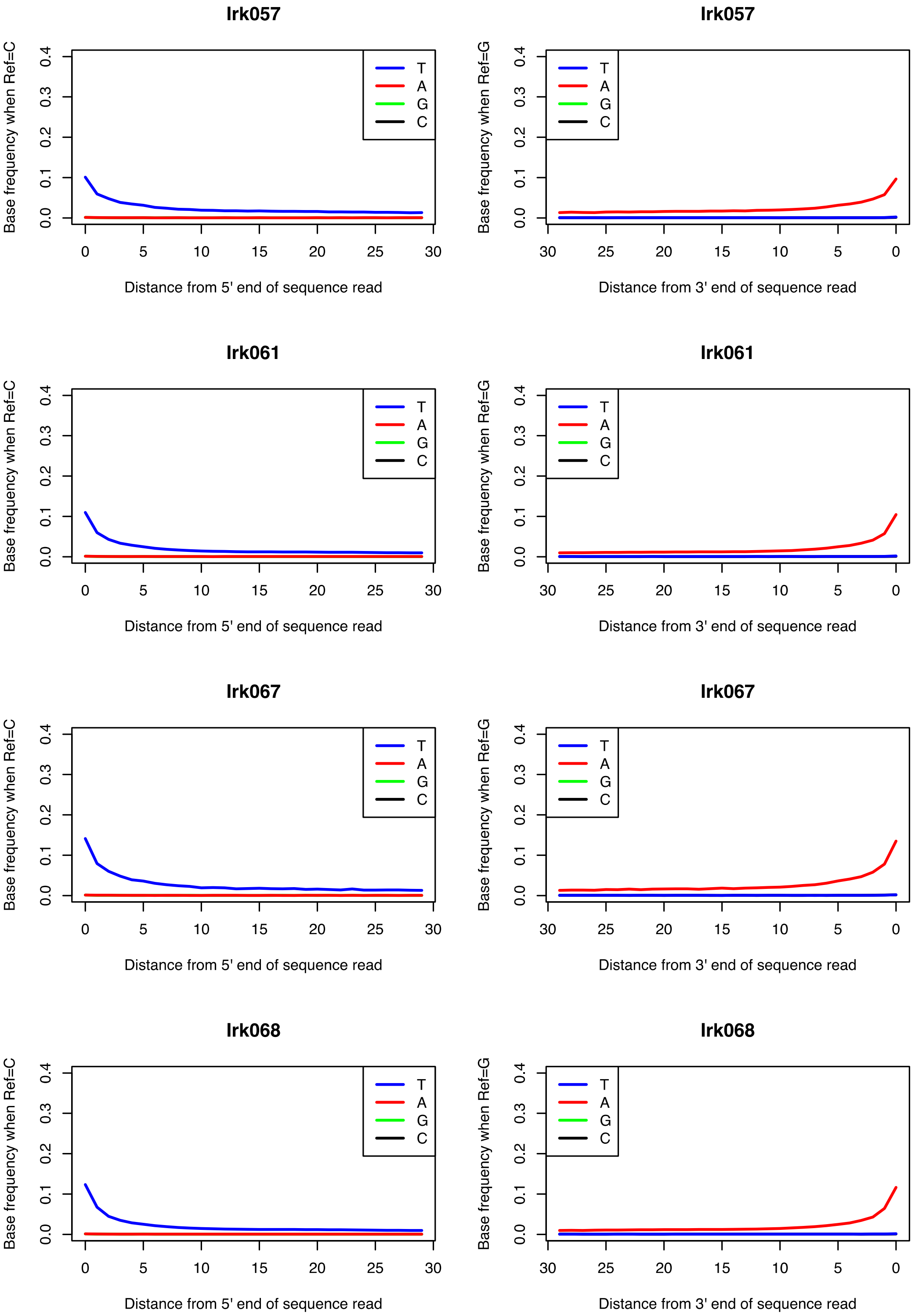

Irk057
Irk057
Irk061
Irk061
Irk067
Irk067
Irk068
Irk068
Base frequency when Ref=C
Base frequency when Ref=G
T
A
G
C
0.0
0.1
0.2
0.3
0.4
0
5
10
15
20
25
30
Distance from 5' end of sequence read
Distance from 3' end of sequence read

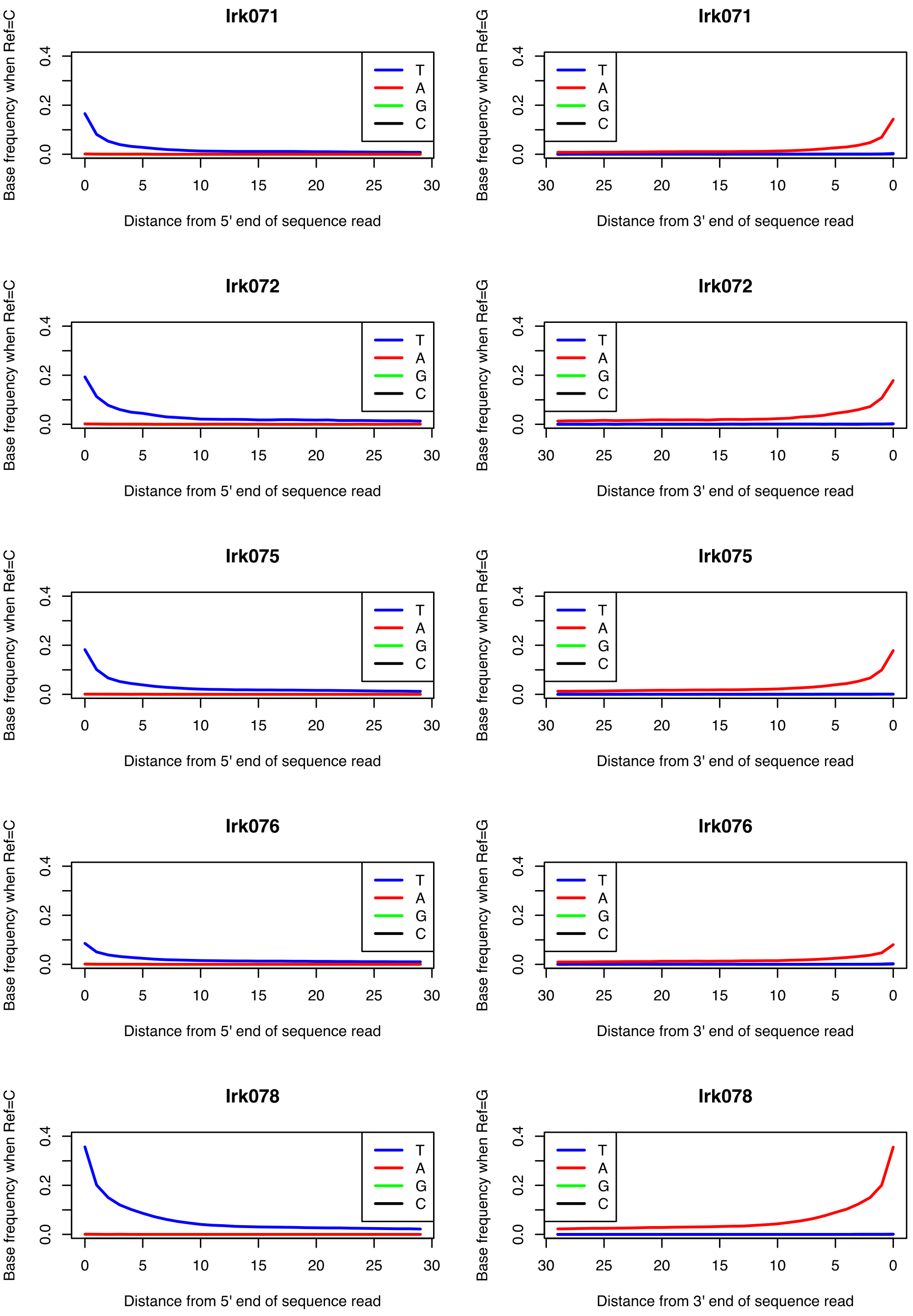

Supplementary Figure S1. Deamination patterns for all individual genome sequences. All show elevated C/T frequencies except N4b2 for which the genome sequence data was generated from damage-repair libraries.

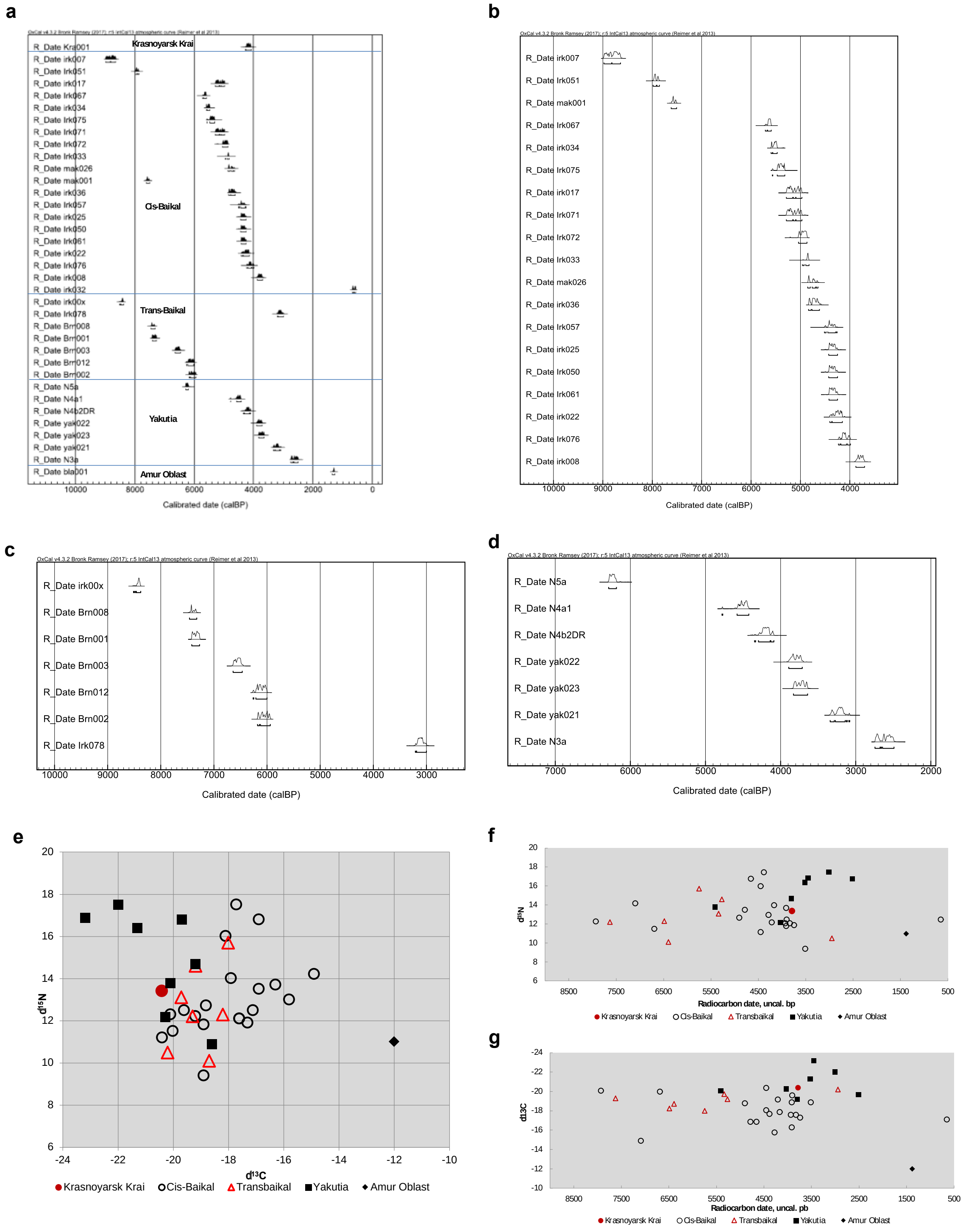

Supplementary Figure S2. The results of the calibration of radiocarbon dates stable isotope analyses of δ^{13}C and δ^{15}N. Calibration for radiocarbon dates was performed with OxCal 4.3.2 (Bronk Ramsey 2009) using the calibration curve IntCal13 atmospheric curve (Reimer et al. 2013). Radiocarbon dates for **(a)** All samples, **(b)** Cis-Baikal samples, **(c)** Trans-Baikal samples, **(d)** Yakutia samples, **(e)** stable isotope

analyses of δ^{13}C and δ^{15}N, **(f)** δ^{15}N vs uncalibrated date, **(g)** bottom δ^{13}C vs uncalibrated age. Sample yak025 excluded.

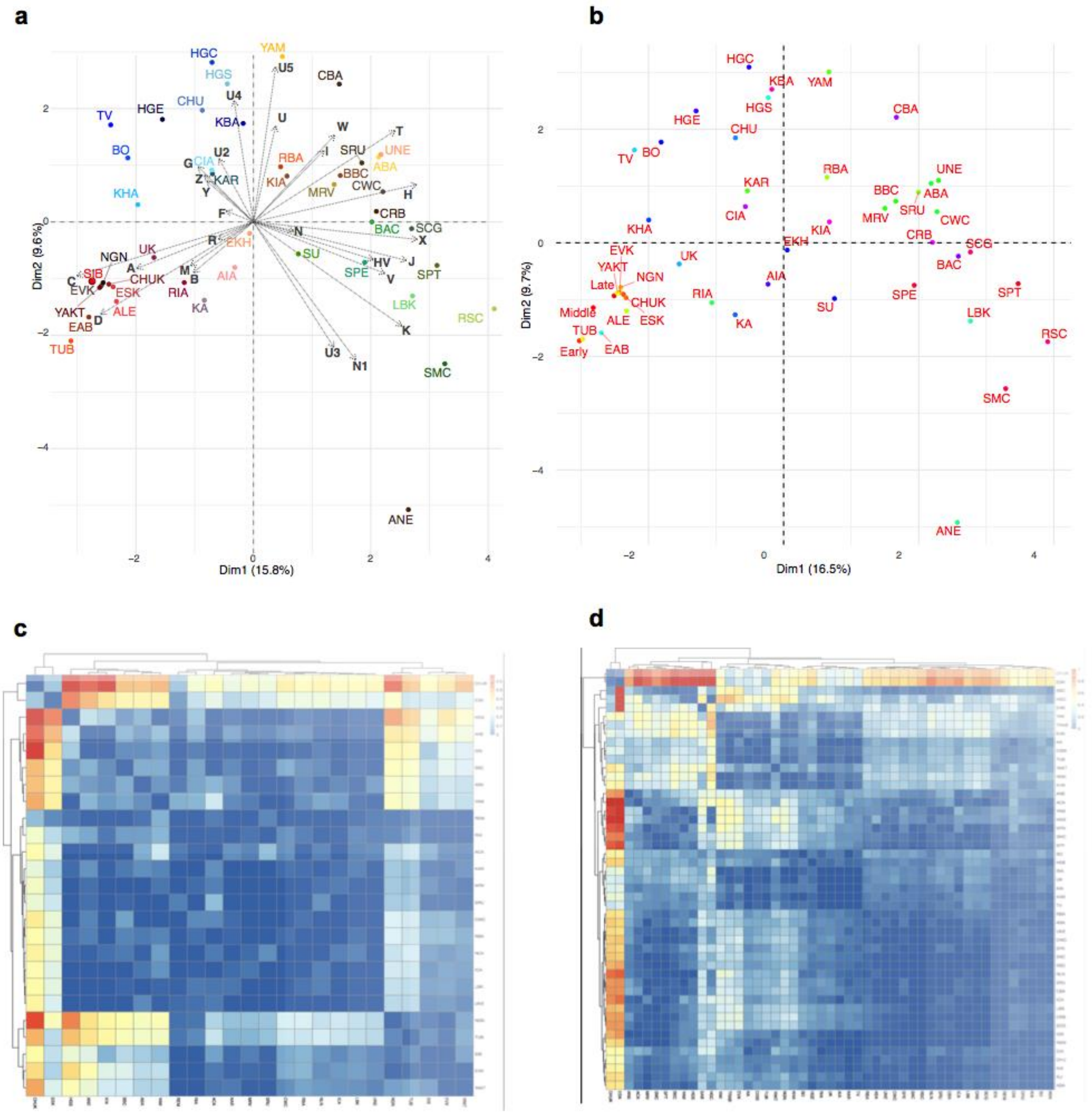

Supplementary Figure S3. Relationship between ancient North Asians and other ancient and present-day populations. Abbreviations of modern groups are CHUK, Chukchi; ESK, Eskimo, EVK, Evenk; NGN, Nganasan; TUB, Tubalar; YAKT, Yakutsk; ALE, Aleut. Abbreviations of ancient groups are RBA, Russia Bronze Age; SRU, Srubnaya; YAM, Yamnaya; KAR, Karasuk, MRV, Hungary Bronze Age; UNE, Unetice, BBC, BellBeaker; CWC, CordedWare; ABA, Armenia Bronze Age; RIA, Russia Iron

Age; ANE, Anatolia Neolithic; LBK, Linear Pottery; EAB, East Asia Bronze Age; TV, Tuva; UK, Ukok; CHU, Chuya; KA, Kazakh Altai; KHA, Khakassia; EKH, ZevakinoChilikta; SU, Southern Ural Sarmatian; BO, Early Bronze Age Russia; AIA; Altai Iron Age; BAC, Baalberge Middle Neolithic; CBA, Central Asia Bronze Age; CIA, Central Asia Iron Age; CRB, Crete Bronze Age; KIA, Kurgan Iron Age; KBA, Kurgan Bronze Age; RSC, Rossen Early Neolithic; SCG, Schöningen Early Neolithic; SMC, Salzmünde Middle Neolithic; SPE, Spain Cardial; SPT, Spain Treilles; HGS; hunter-gatherers; HGC, Central European hunter-gatherers; HGE, East European hunter-gatherers. **(a)** PCA plot based on haplogroup frequencies calculated using HVRI mitochondrial sequences. Ancient North Asians from Siberia were plotted as a single group (SIB, n=41). (**b)** PCA plot based on haplogroup frequencies calculated using HVRI mitochondrial sequences. Ancient North Asians from Siberia were grouped as three temporal groups. **(c)** Fst between 41 ancient North Asians grouped into three spatial populations and published ancient and modern groups with full mitochondrial sequences. **(d)** F_{st} between 41 ancient North Asians grouped into three spatial populations and published ancient and modern groups with HVRI sequences.
